# Neonatal diethylstilbestrol exposure disrupts uterine epithelial apical–basal polarity and partial EMT state

**DOI:** 10.1073/pnas.2603458123

**Published:** 2026-08-17

**Authors:** Rachel E. Bainbridge, Wendy N. Jefferson, Tianyuan Wang, Sara A. Grimm, Carmen J. Williams

**Affiliations:** ^a^ Reproductive and Developmental Biology Laboratory, National Institute of Environmental Health Sciences, NIH, Durham, NC 27709; ^b^ Biostatistics and Computational Biology Branch, National Institute of Environmental Health Sciences, NIH, Durham, NC 27709

**Keywords:** cell–cell communication, endocrine disruption, endometrium, multiomic sequencing, stem cells

## Abstract

The developing female reproductive tract is highly sensitive to external hormonal stimulation, which can result in infertility and gynecologic diseases. To determine the underlying mechanisms, we used a mouse model to test the direct, cell type–specific effects of neonatal exposure to the estrogenic chemical, diethylstilbestrol (DES), on the developing uterus. We found that control uterine epithelium is in a partial epithelial–mesenchymal transition state that is lost following DES exposure. This is accompanied by evidence of premature differentiation including altered apical–basal cell polarity and absence of the *Lgr5*+ epithelial stem cell population required for uterine gland formation. Cell–cell communication between epithelial and mesenchymal cells is restructured, and Wnt signaling is aberrantly activated in the epithelium. The DES-exposed uterine mesenchyme has early signs of fibrosis through increased deposition of extracellular matrix (ECM) collagen. Mechanistically, DES exposure causes cell type–specific changes in chromatin accessibility and gene expression, most prominently in epithelial cells. These changes can be explained in part by cell-specific alterations in chromatin looping at enhancer regions in concert with alterations in ERα binding. These findings suggest that reprogramming cell type–specific differentiation trajectories and ECM characteristics underlie the long-term phenotypic effects of developmental exposure to DES and possibly other estrogenic endocrine disrupting chemicals. These changes lead to functional impairment of adult tissues and increased cancer risk.

## Significance

Uterine development is strongly impacted by brief exposure to estrogenic endocrine disruptors, but it is unclear what mechanisms explain why development is such a sensitive time point. This study employed multiomic analysis to identify cell type–specific uterine developmental trajectories in neonatal mice exposed to the estrogenic chemical, diethylstilbestrol, and compared these to controls. Control epithelium was under the influence of carefully orchestrated Wnt/β-catenin signaling and was in a partial epithelial-to-mesenchymal transition state. Diethylstilbestrol (DES) exposure activated Wnt/β-catenin signaling and drove the epithelium toward differentiation, resulting in the loss of both epithelial stem cells and normal apical–basal polarity. These changes provide an explanation for how endocrine disruptors can divert intrinsically programmed developmental trajectories to alter adult organ function.

Exposure to estrogenic endocrine disrupting chemicals during development causes an increased risk of infertility and cancer in women and in animal models (1). A classic example was the prenatal exposure of millions of women to the xenoestrogen, diethylstilbestrol (DES), which caused structural and functional reproductive tract anomalies and associated pregnancy complications. In addition, the exposed women are at higher risk for vaginal clear cell adenocarcinoma, uterine fibroids, endometriosis, and breast cancer (2). Developmental endocrine disruption has distinct effects compared to adult responses to the same exposures, a difference likely explained by increased cellular plasticity during development and the impact on the epigenetic state of the tissue. However, the mechanistic underpinnings for how these changes are induced and then maintained over time are not known.

In humans and rodents, the female reproductive tract develops structurally prior to birth; however, cellular differentiation occurs over a much wider time span. In both species, uterine gland differentiation continues until around puberty (3, 4). This developmental trajectory means that the uterus has increased susceptibility to environmental influences through puberty. Indeed, women exposed as infants to soy based infant formulas, which contain high levels of the phytoestrogen, genistein, have an increased incidence of uterine fibroids and endometriosis (5, 6). In mice, neonatal exposure to either DES or genistein causes altered cellular differentiation, infertility, and cancer (7–11). These phenotypes are accompanied by alterations in rostral-caudal patterning, a variable degree of failure of uterine gland development, and epithelial differentiation abnormalities (basal cell and squamous metaplasia) in addition to adenocarcinoma (12–16). Neonatal exposure to a high dose of DES (1 mg/kg/d) results in ~90% uterine cancer incidence, providing a very robust, highly penetrant mouse model to study how these types of exposures alter differentiation and the epigenome to cause altered adult function and cancer (10).

A partial epithelial–mesenchymal transition (EMT) that results in hybrid cell type states frequently occurs during tissue morphogenesis (17). This partial EMT is characteristic of processes as diverse as gastrulation and mammary gland branching morphogenesis; it is also observed in invasive and metastatic cancer cells (18). Cellular features of partial EMT vary widely but can include alterations in apical–basal polarity and cell adhesion to extracellular matrix (ECM) components. These changes are often accompanied by maintenance of classical epithelial features: stable epithelial junctional complexes linked to cytoskeletal elements that support interepithelial cell adhesion (18). Endometrial gland development follows a trajectory similar to that of mammary glands, with budding of epithelial cells into the underlying mesenchyme followed by cell proliferation and continued extension to create the final gland structures (19). In the mouse, this process begins around postnatal day 8 (PND8) and requires expression of the Wnt signaling regulator, LGR5, which also serves as an epithelial stem cell marker (20). There is evidence in adult uterine tissue that estrogen exposure can repress *Lgr5* gene expression (21), but whether or not this occurs during neonatal uterine differentiation is unknown. In addition, cell type–specific impacts of DES or other estrogenic chemicals on uterine epithelial cell transition states are unknown.

Estrogen actions in the mouse uterus are largely mediated by estrogen receptor alpha (ERα) interactions with chromatin to modulate gene expression (22). ERα binds chromatin in the absence of ligand but changes its binding sites and binding partners following ligand activation (23). In adult mice undergoing regular estrous cycling, changes in steroid hormone levels across the cycle modulate ERα binding sites and three-dimensional chromatin structure, along with changing chromatin accessibility and enhancer switching (24). In this setting, the progesterone receptor (PGR) is a key binding partner for ERα. How ERα influences gene expression in the neonatal mouse uterus and whether it interacts at that time of development with PGR is not known. On PND1, *Pgr* is not expressed except sporadically, but its expression rapidly increases through PND5, suggesting that PGR protein is likely present for at least some of this time and could influence ERα-mediated chromatin dynamics (25).

To explore the immediate effects of DES exposure on uterine cell identity, we performed single nucleus multiomic sequencing, which produces paired RNA-seq and ATAC-seq data from each cell, on PND5 uteri. We used these data to evaluate cell type–specific signaling interactions and validated some of these at the protein level. Our key findings were that the neonatal uterine epithelium is normally in a partial EMT state and that premature differentiation accompanied by alterations in apical–basal polarity and loss of epithelial stem cells are key cellular features explaining DES-induced adult uterine phenotypes. Identification of ERα binding sites and three-dimensional chromatin structure in bulk PND5 uterine tissue provides a chromatin-centric view of how DES alters cell differentiation trajectories to interfere with proper uterine development and growth.

## Results

### DES Exposure Induces Gene Expression Changes Largely in Epithelial and Mesenchymal Cells.

To explore cell-type specific impacts of neonatal DES exposure on gene expression and chromatin accessibility in early uterine development, we performed single-nucleus multiomic sequencing of the uterus of DES-exposed or control mice on PND5. From control uteri, 20,000 cells (maximum cutoff) were obtained with 2,571 median genes per cell and 1,229 high-quality ATAC fragments per cell. From DES-exposed uteri, 13,164 cells were obtained with 1,473 median genes per cell and 3,335 high-quality ATAC fragments per cell. Following initial quality control, 13,154 cells from control uteri and 7,339 cells from DES-exposed uteri were used for analysis (*SI Appendix*, Fig. S1*A*).

**Fig. 1.**
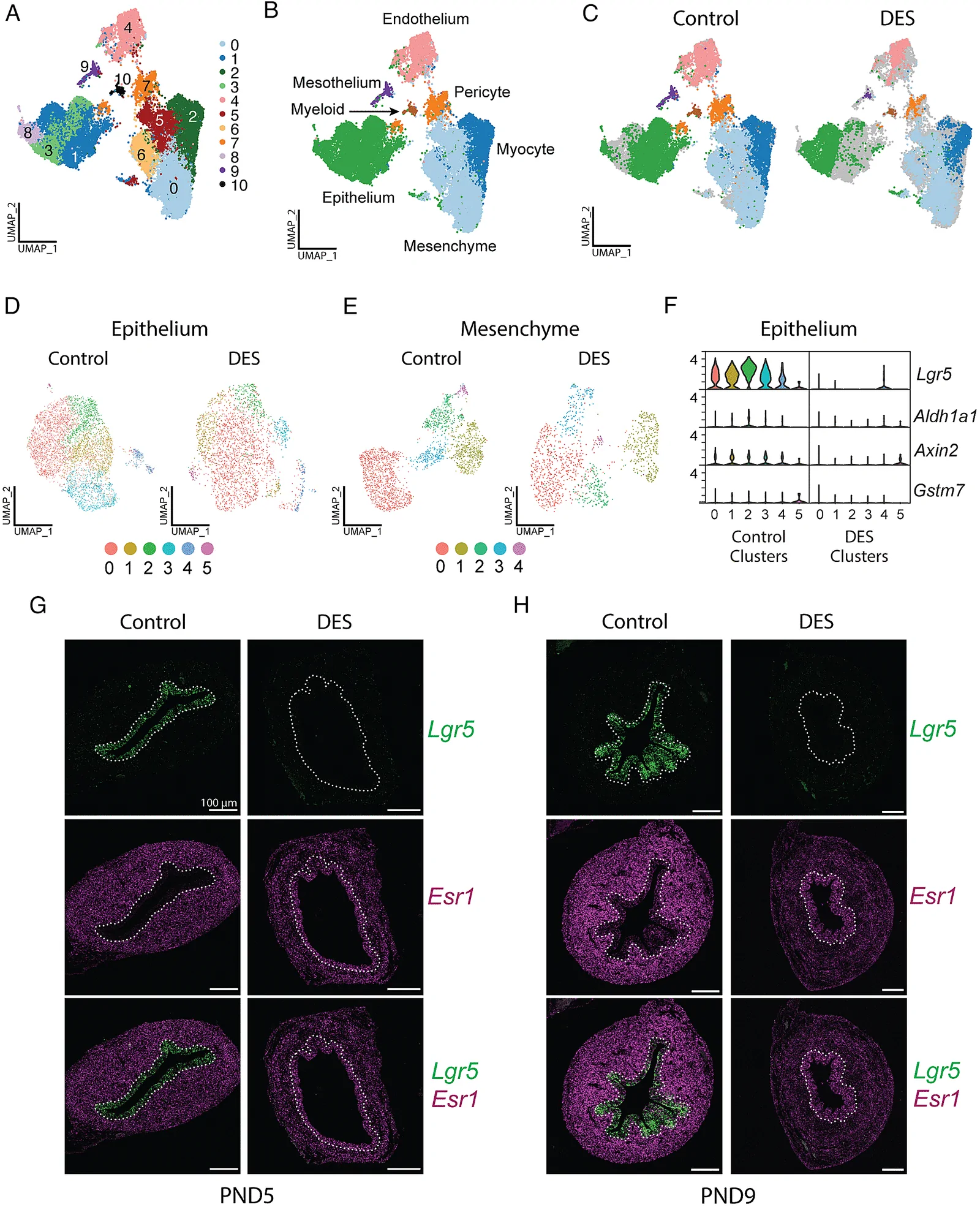
Single-nucleus RNAseq demonstrates differences in gene expression between control and DES-exposed uterine mesenchyme and epithelium. (*A*) Integrated UMAP of all cells captured with snRNAseq from control or DES-exposed uteri labeled by cluster. (*B*) Integrated UMAP with manually annotated cells including mesenchyme, epithelium, myocyte, endothelium, pericyte, mesothelium, and myeloid cells. (*C*) Annotated UMAP split by treatment. (*D* and *E*) UMAPs of cells from control or DES-exposed epithelium (*D*) and mesenchyme (*E*). (*F*) Violin plots of stem cell marker genes *Lgr5, Aldh1a1, Axin2*, *Gstm7* in control or DES-exposed epithelium. (*G* and *H*) Representative images of cross sections of PND5 (*G*) or PND9 (*H*) uterine horns from control or DES-exposed mice, with fluorescent RNA probes for *Lgr5* (*Top*, green) and *Esr1* (magenta, *Middle*; overlay, *Bottom*). The epithelium is outlined with dotted lines. PND5 Control, N = 15 from 3 to 7 mice; PND5 DES, N = 10 from 3 to 6 mice; PND9 Control, N = 9 from 4 mice; PND9 DES, N = 4 from 4 mice.

To determine differences in uterine cell type composition, we assessed cell identity in an integrated dataset of both DES-exposed and control cells using the single nucleus RNA sequencing (snRNAseq) data. An integrated UMAP (uniform manifold approximation and projection) of all captured uterine cells showed 11 distinct clusters (Fig. 1*A* and Dataset S1). We performed manual cell identity annotation using markers published in a single cell RNAseq dataset from C57BL/6J uteri at PND3 (26) (*SI Appendix*, Fig. S1*B*). To determine if use of both snRNAseq and ATACseq data would improve cell type identification, we performed weighted-nearest neighbor analysis (27). Nearly 95% of cells were assigned the same cell type as that based only on RNA (*SI Appendix*, Fig. S1*C*), so the RNA-based cell identity assignments were used for all later analyses. Cells clustered mainly by cell identity (Fig. 1*B*). The only obvious difference in cell clusters between exposure groups was the epithelial cells, which were generally nonoverlapping between control and DES (Fig. 1*C*). Though there were fewer cells within each DES-exposed cell identity group, the relative proportions of each were similar between DES-exposed and control uteri, except for mesenchyme, pericytes, and endothelium (*SI Appendix*, Fig. S1*D*). Gene expression across cell types and in the two exposure groups was compared by generating a heatmap of the top 25 differentially expressed genes (DEGs) per cell type and exposure (*SI Appendix*, Fig. S2*A* and Dataset S2). DES-exposed epithelial and mesenchymal cells had overall lower expression of the control markers and expressed unique marker genes. This phenomenon was not observed in the remaining cell types, which had similar levels of marker gene expression in both exposure groups.

DEGs between DES and control were determined for each cell type (Dataset S2; 18,038 genes across all cell types). These were compared to the list of DEGs from our published PND5 bulk uterine RNAseq dataset from mice exposed to DES under the same protocol (28) (7,046 genes; adjusted *P*-value < 1 × 10^−5^). Many DEGs were identified by snRNAseq in one or more of the uterine cell types that were not identified in the bulk RNAseq (4326/8395, 51.5%) (*SI Appendix*, Fig. S2*B*). In addition, many genes also were uniquely differentially expressed in the bulk RNAseq that were not captured by snRNAseq (2799/7046, 42.3% of bulk DEGs; *SI Appendix*, Fig. S2*B*); most of these DEGs exhibited lower gene expression than genes that overlapped snRNAseq DEGs (*SI Appendix*, Fig. S2*C*). These observations demonstrate the differential utility of both approaches, with cell specificity provided by one approach and greater ability to detect less abundant RNAs by the other. The majority of the snRNAseq DEGs were differentially expressed in both epithelium and mesenchyme, with about half of these also DEGs in endothelium.

To explore DES-induced changes in gene expression in epithelial and mesenchymal cells, we restricted the integrated whole uterine dataset to only epithelial cells and mesenchymal cells. Because myocytes clustered closely with mesenchymal cells, with some myocytes overlapping the mesenchymal cluster, myocytes were also included with mesenchymal cells. Analyzing only this cell subset retained the large majority of cells while eliminating the small, more specialized cell populations (*SI Appendix*, Fig. S1*D*), simplifying the analysis and resulting in 6,678 DEGs (Dataset S3). However, in the resulting UMAP, clusters were based almost entirely on exposure status, masking any cell type–specific differences between populations (*SI Appendix*, Fig. S3*A*). An overlap of these DEGs with estradiol-regulated genes (fold change ≥ |1.5|, FDR ≤ 0.05) in uteri of ovariectomized mice, from a published microarray dataset (29), identified 29% of the 6,678 DEGs as estradiol-regulated. We interpret these data to indicate that estrogen regulation is the main driving factor in separation of control and DES-exposed cells in UMAP space.

To allow better identification of cell type–specific differences, the epithelial/mesenchymal/myocyte dataset was split by exposure status and each exposure group analyzed separately (*SI Appendix*, Fig. S3 *B* and *C*) (15). In both control and DES UMAPs, cells were defined largely by epithelial or mesenchymal characteristics (*SI Appendix*, Fig. S3 *D*–*G* and Datasets S4 and S5). Within the mesenchymal population, though cells most strongly expressed mesenchymal markers, they also expressed either stromal (*Vcan*, *Dcn*) or myocyte (*Myh11, Chrm3*) markers at a lower level (*SI Appendix*, Fig. S3 *D* and *E*) (15, 26, 30). Epithelial and mesenchymal (stroma-like, but not myocyte-like) populations were subset from these datasets and used in subsequent analyses to explore differences within each cell type due to DES exposure. Both control and DES-exposed epithelial cells (Fig. 1*D*) and control and DES-exposed mesenchymal cells (Fig. 1*E*) formed discrete clusters (epithelial, 6 clusters; mesenchymal, 5 clusters). DES exposure resulted in 2,603 upregulated and 4,028 downregulated genes in epithelial cells (Dataset S6) and 1,189 upregulated and 1,987 downregulated in mesenchymal cells (Dataset S7), which were further explored.

### PND5 DES-Exposed Epithelial Cells Lack a Typical Stem Cell Population.

We demonstrated previously that epithelial stem cells could not be identified in the uterus of 12-month-old adults exposed neonatally to DES (15). To determine if this population was lost with age or was never properly established, we examined markers of stem cell populations at PND5. Control PND5 epithelial cells expressed classic stem cell markers, including *Lgr5*, *Aldh1a1*, *Axin2*, and *Gstm7*; *Lgr5* exhibited the highest expression (Fig. 1*F*) (15). The highest expression of *Lgr5* was observed in control cluster 2, but it was also expressed in most cells of all other clusters (Fig. 1*F*). The stem cell markers were minimally or not expressed in DES-exposed epithelium (Fig. 1*F* and *SI Appendix*, Fig. S3*H*).

To validate the *Lgr5* expression using a second methodology, we performed in situ hybridization on cross-sections of uterine tissue. *Esr1* was used as a positive control for the method as the protein is detected in the neonatal female reproductive tract (31, 32). In PND5 controls, *Lgr5* was detected in most epithelial but not mesenchymal cells, while *Esr1* was detected only in mesenchymal cells (Fig. 1*G*). In DES-exposed epithelium, *Lgr5* was not detected but *Esr1* was expressed in both epithelium and mesenchyme. These findings are consistent with previous reports of loss of uterine *Lgr5* expression following estradiol treatment of ovariectomized adults (21). To determine if the DES-induced loss of *Lgr5* expression persisted to the time when gland formation begins, we performed in situ hybridization on control and DES-exposed uteri at PND9. *Lgr5* was readily detected in control epithelium but not in DES-exposed uteri (Fig. 1*H*). *Esr1* was minimally expressed in the epithelium and highly expressed in the mesenchyme in control uteri. DES-exposed uteri continued to have *Esr1* expression in epithelial cells and reduced levels in mesenchyme compared to controls on PND9 (similar levels to PND5 control mesenchyme) suggesting failure to up-regulate *Esr1* in DES-exposed mesenchyme. These data support the idea that neonatal DES exposure induces loss of the existing stem cell population after only 5 days of treatment.

### DES-Exposure Alters Cell–Cell Communication in the Developing Uterus.

The communication between mesenchyme and epithelium is vital for proper reproductive tract development (31, 33, 34). To explore the global capacity of these cell types to communicate following DES exposure, we assessed expression of known ligand–receptor pairs using the R package, LIANA (35). The relative number of all ligand–receptor pairs in both epithelium and mesenchyme in control and DES uteri is shown as a chord map (*SI Appendix*, Fig. S4*A*). Control and DES cells had similar percentages of mesenchyme-to-mesenchyme signal pairs but differed in all other signal pair patterns. Mesenchyme to epithelium signaling predominated in control cells, while epithelial to mesenchyme signaling predominated in DES cells. Epithelium-to-epithelium signaling pairs were present in controls, but the small number of such pairs in DES cells was insufficient to visualize in the chord map.

**Fig. 2.**
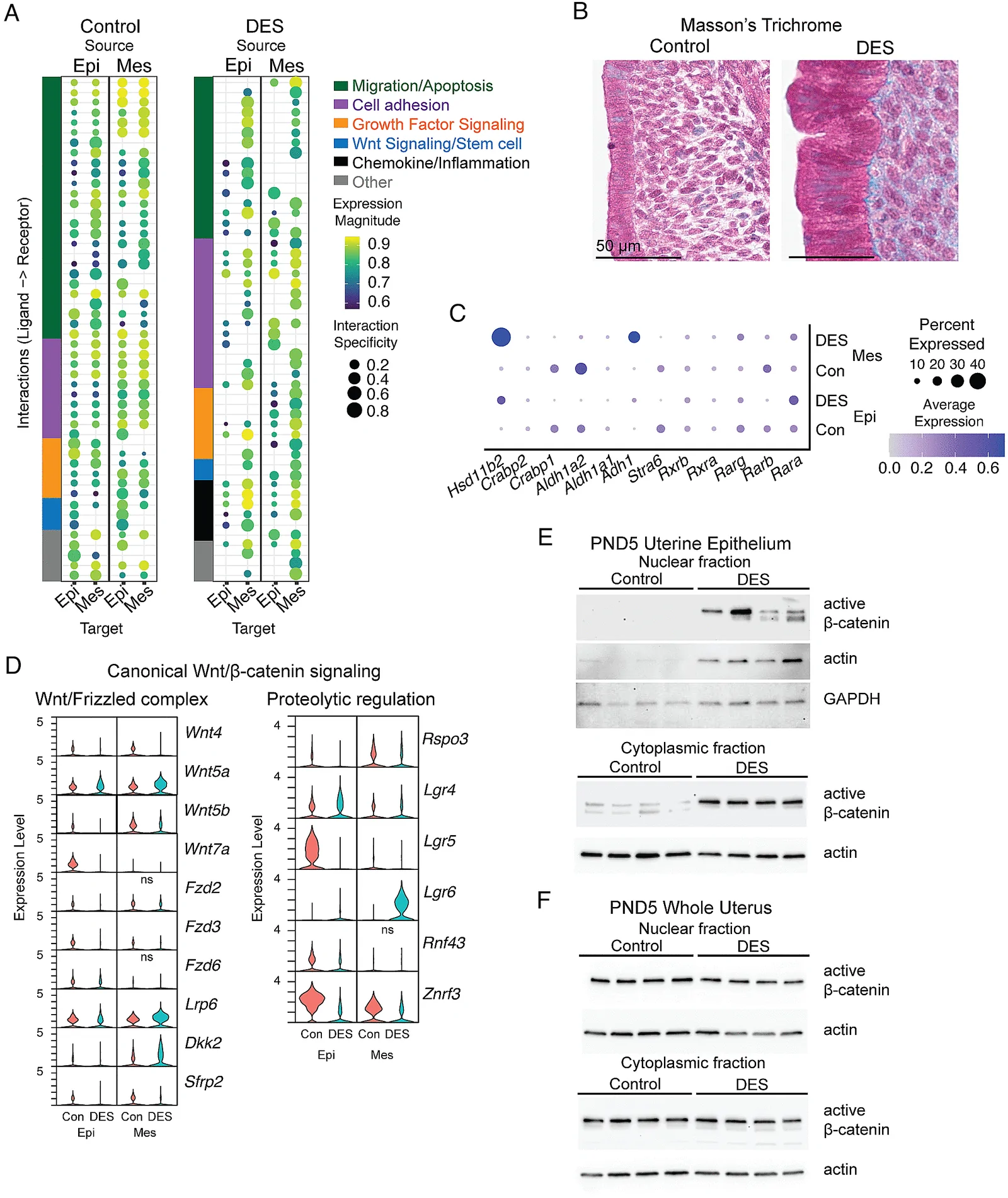
DES exposure alters uterine epithelial–mesenchymal communication and activates epithelial Wnt/β-catenin signaling. (*A*) Dot plots representing the top 50 ligand–receptor pairs available to interact (by presence of RNA) in control or DES-exposed epithelium or mesenchyme. Label at top notes ligand source; label at bottom notes target receptor source. Each row represents a ligand–receptor pair, clustered and color coded to denote function. Ligand–receptor pairs included are listed in Dataset S8. (*B*) Representative Masson’s trichrome staining of cross sections of PND5 uterine horn from control or DES-exposed mice (blue denotes collagen; Control, N = 11 from 7 mice; DES, N = 12 from 6 mice). (*C*) Dot plot of retinoic acid signaling gene expression in control or DES-exposed epithelium and mesenchyme. (*D*) Violin plots of Wnt/β-catenin complex members in control or DES epithelium or mesenchyme. Gene expression in all violin plots is significantly different unless marked as “ns.” (*E* and *F*) Representative images of active β-catenin immunoblots of PND5 control or DES-exposed uterine epithelial (*E*) or whole uterine (*F*) nuclear or cytoplasmic fractions, as indicated. The corresponding immunoblots for actin and/or GAPDH are shown. For *E*, epithelium was pooled from 5 uteri per sample from control and DES-exposed mice (N = 4 samples per group). For *F*, each sample is from an individual uterus from control and DES-exposed mice (N = 4 per group).

To examine the functions of the predicted intercellular signaling pairs, we plotted the top 50 ligand–receptor pairs from each condition by rank aggregate score (Fig. 2*A* and Datasets S8–S10). These ligand–receptor pairs were grouped by function to reveal patterns in functional gains or losses in communication. In controls, migration and/or apoptotic signaling pathways had the largest number of ligand–receptor pairs. [Note that migration and apoptosis are grouped in this analysis as many of these pairs act in shared pathways that balance the two processes (36)]. Additional pathways well represented in controls were growth factor signaling and cell adhesion. The pairs represented in these three pathways generally had ligands and receptors in both epithelium and mesenchyme, suggesting active communication within and between both cell types. These findings differ somewhat from a previous analysis of ligand–receptor pairs in PND3 uteri in that growth factor signaling (TGFB, FGF, IGF, and BMP signaling) in that case was identified as very significant but was largely directed from mesenchyme to epithelium rather than bidirectional, and cell adhesion pathways were not identified (37). Control cells had only 3 ligand receptor pairs in the Wnt signaling pathway, and these were different in that the ligands were expressed in both epithelium and mesenchyme, but the receptors were only in the epithelium. This finding indicates that Wnt signaling in controls is directed toward epithelial cell responses, very similar to the previous report (37). Pairs annotated as “other” had assorted functions that did not fit into a larger group with shared function.

In DES cells, the most well represented pathways were similar to controls, however, there were far fewer pairs in migration/apoptosis and far more in cell adhesion pathways (Fig. 2*A*). In addition, rather than ubiquitous cell type communication predicted in controls, there was minimal predicted epithelium-to-epithelium communication in DES cells (*SI Appendix*, Fig. S4*A*). The essential Wnt signaling pathway was represented by two ligand–receptor pairs. However, there was no epithelium-to-epithelium Wnt signaling and the critical mesenchyme-to-epithelium signaling was reduced. Unlike in controls, there was generally lower expression of growth factor signaling ligands in epithelium (Fig. 2*A* and Datasets S8 and S9), consistent with previous observations of a dramatic reduction in cell proliferation following DES exposure (12, 13, 38). However, DES-exposed mesenchyme had consistently high expression magnitude of mesenchymal growth factor ligands paired with mesenchymal targets (Fig. 2*A* and Datasets S8 and S10). The highest ranked pair in this set was *Tgfb2*:*Tgfbr3*, important regulators of fibrosis and ECM production (39). In addition, a distinct signaling pathway, chemokine/inflammation, was represented by six ligand–receptor pairs in the DES cells but none of these were observed in controls. Overall, DES-exposed uteri contained epithelial cells that were less communicative with each other, less receptive to mesenchymal signals, and most likely received more adhesive and inflammatory signals and fewer growth and migratory signals than controls. Taken together, these findings suggest that DES-exposed epithelial cells have a reduced ability to perform normal growth directed signaling and that DES-exposed mesenchymal cells are activated to create a fibrotic milieu.

Many of the predicted upregulated cell adhesion ligands were collagens, which are key ECM components. This finding is consistent with previous observations of increased ECM in PND7 and adult uterine stroma following neonatal exposure to estrogenic chemicals, including DES, genistein, and bisphenol A (14, 38, 40). The most highly expressed collagens were fibril-forming types I, III, and V, with two type I isoforms (*Col1a1* and *Col1a2*) and two type V isoforms (*Col5a1* and *Col5a2*) highly upregulated following DES exposure in mesenchyme; the type I isoforms were also upregulated in epithelium (*SI Appendix*, Fig. S4*B*). Genes encoding type IV collagen, a major component of basement membranes, were generally downregulated following DES exposure in both epithelium and mesenchyme. Multiple type VI collagen isoforms, which form beaded filaments rather than fibrils, were also highly upregulated in the mesenchyme following DES exposure. Consistent with these observations, Masson’s trichrome staining revealed that collagen was excessively accumulated in DES uterine mesenchyme compared to controls (Fig. 2*B*). There was also very intense red staining of the DES epithelial cells, suggesting increased levels of keratins. This finding was consistent with the dramatically increased expression of *Krt7*, *Krt8*, and *Krt19* in DES epithelium (Dataset S6). Matrix metalloproteases degrade collagen in mesenchyme, which supports epithelial invasion during gland development (41–43). In controls, genes encoding matrix metalloproteases (*Mmp2*, *Mmp14*, and *Mmp16*) were expressed in epithelium and at a higher level in mesenchyme; these changes were accompanied by expression of tissue inhibitor of metalloprotease 3 (*Timp3*) (*SI Appendix*, Fig. S4*C*). Expression of all these genes was decreased with DES exposure, suggesting that both increased collagen deposition and reduced matrix metalloprotease expression contribute to alterations in the ECM character of the DES-exposed uterus at PND5.

Retinoic acid signaling is implicated in the mesenchymal–epithelial crosstalk that orchestrates development of the female reproductive tract. In adult mice, retinoic acid signaling limits the impact of estrogen signaling on the uterine epithelial phenotype, helping to maintain it as a simple columnar rather than stratified epithelium (44). There is also evidence for the converse, that estrogen signaling in the uterus may limit retinoic acid signaling (44). In the current study, retinoic acid signaling mediators were not identified as ligand–receptor pairs in either control or DES uteri (Datasets S9 and S10). Although both epithelial and mesenchymal cells expressed some retinoic acid signaling receptors, mediators, and targets, these were detected in very few cells and at a low expression level in either compartment (Fig. 2*C*). An exception was *Hsd11b2*, a retinoic acid signaling target that was upregulated, rather than downregulated, in the mesenchyme following DES exposure. These findings suggest that the PND5 uterus is not yet relying on retinoic acid signaling to limit estrogen effects, consistent with the lack of endogenous estrogen at this time in development.

### DES Exposure Activates Epithelial Wnt Signaling.

The Wnt/β-catenin signaling pathway is important for regulating growth and development, with nuclear translocation of β-catenin and interaction with TCF/LEF transcription factors (TFs) key for its transcriptional activity (45, 46). Following neonatal DES exposure, expression of Wnt signaling ligands, receptors, and targets is dramatically increased in uterine epithelial cells at 12 mo of age (15), but it was unknown when this increased activity began. Neonatal DES exposure suppresses many Wnt signaling mediators (*Wnt4*, *Wnt7a*, *Wnt11*, *Wnt16*, and *Fzd10*) though it increases *Wnt5a* in the PND5 uterus (12, 13). How these changes affect overall Wnt signaling leading to transcriptional responses is unknown. To determine at a cell type–specific level how Wnt signaling was likely to be impacted immediately following neonatal DES exposure, we generated violin plots of the expressed genes that participate in regulating Wnt signaling at the plasma membrane (Fig. 2*D*). In PND5 control epithelium, the most strongly expressed components of signaling directly at the frizzled receptor complex were the activating ligands, *Wnt5a* and *Wnt7a*, the inhibitory ligand, *Sfrp2*, and transmembrane components, *Fzd3*, *Fzd6*, and *Lrp6*. Components of the plasma membrane complex responsible for ubiquitinating frizzled receptors were also expressed strongly, including *Lgr5* and *Znrf3*. *Rspo* genes, which encode the ligands that promote downregulation of the LGR/ZNRF3 complex, were not expressed in the epithelium. Control mesenchyme expressed additional secreted regulators that could impact Wnt signaling in the epithelium, including both activators (*Wnt4*, *Wnt5a*, and *Wnt5b*), and inhibitors (*Dkk2* and *Sfrp2*). Overall, this gene expression profile provided a picture of balanced Wnt signaling activation that was tempered by both ligand-based inhibition and proteolytic degradation. The control mesenchyme had similarly balanced Wnt signaling gene expression, with *Fzd2*, *Fzd3,* and *Lrp6* expressed and potentially activated by several expressed Wnts. Proteolytic degradation to counteract this activation would be expected through strong *Znrf3* expression because the absence of *Lgr* gene expression to produce an LGR receptor would prevent RSPO3 from acting to turn over ZNRF3.

In PND5 DES uteri, *Wnt4* and *Wnt7a* were downregulated in the epithelium and *Wnt5a* was upregulated in mesenchyme (Fig. 2*D*). *Fzd6* in epithelium and *Fzd2* in mesenchyme were expressed at low levels, like controls. In addition, genes encoding components of the proteolytic regulation complex were expressed, including *Lgr4*, *Lgr6*, and *Znrf3*. To determine if these differences in RNA expression of Wnt signaling components had functional consequences, we examined PND5 uteri for the presence of nuclear active β-catenin as an indicator of β-catenin-driven transcription. DES epithelium had strongly upregulated active β-catenin in both cellular compartments (Fig. 2*E* and *SI Appendix*, Fig. S5 *A*–*E*). Epithelial nuclear actin was also increased following DES exposure, consistent with findings that β-catenin nuclear localization and transcriptional regulatory activity depend on nuclear actin (47, 48). In whole PND5 uterine tissue, which is composed mainly of mesenchyme, active β-catenin and actin were detected at similar levels in nuclei and cytoplasm of control and DES-exposed tissues (Fig. 2*F* and *SI Appendix*, Fig. S5 *F*–*I*). These findings indicate that DES activates canonical β-catenin-mediated gene expression in uterine epithelium but does not significantly change its activity in mesenchyme.

### DES Exposure Alters Epithelial Apical–Basal Polarity and Partial EMT State.

**Fig. 3.**
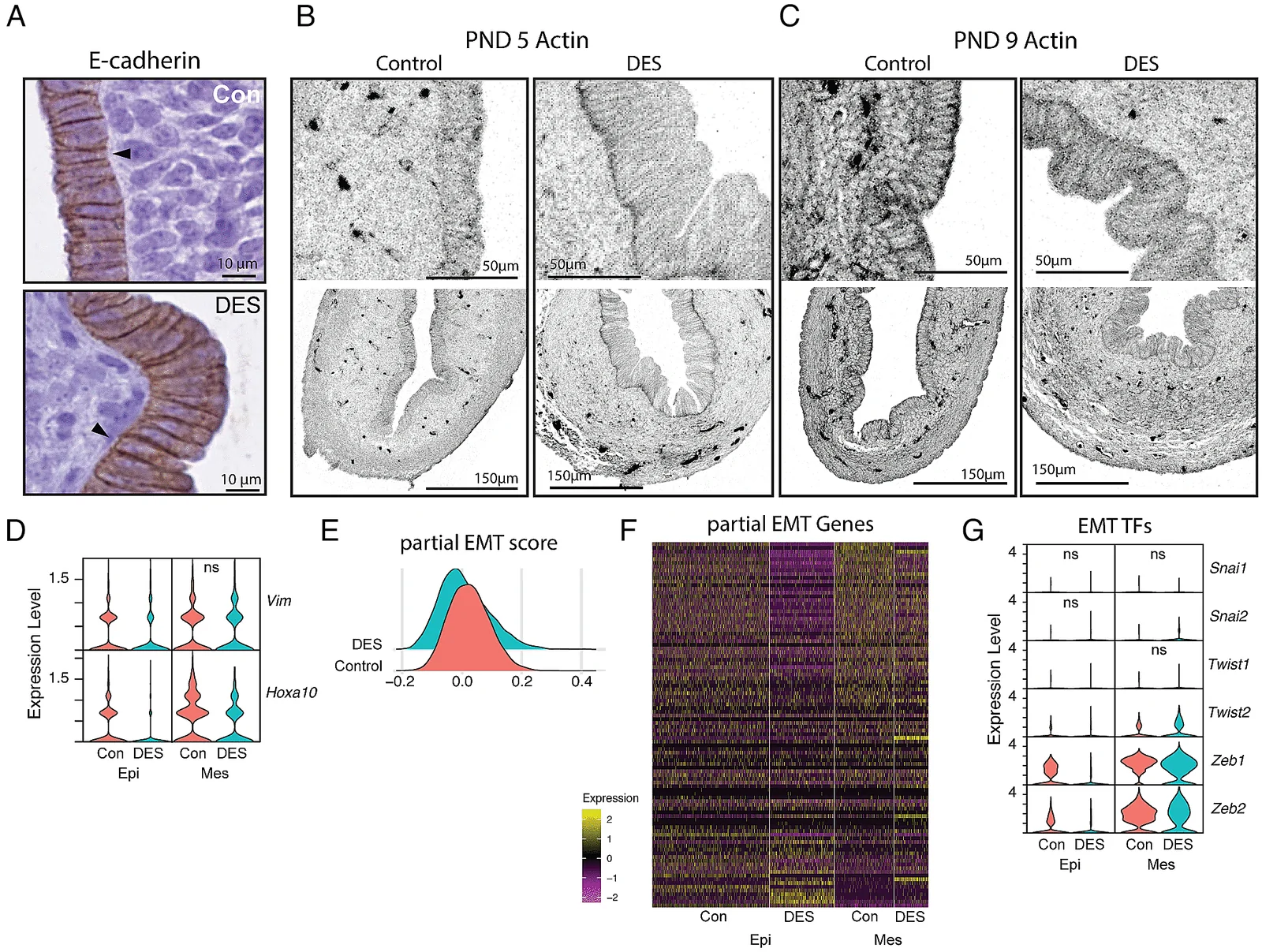
DES exposure alters epithelial cell polarity and EMT state. (*A*) Representative images of E-cadherin immunohistochemistry in control or DES-exposed uterus at PND5 (Control, N = 14 from 3 to 6 mice; DES, N = 12 from 3 to 6 mice). (*B* and *C*) Representative images of filamentous actin staining in PND5 (*B*) or PND9 (*C*) control or DES-exposed uterus (Control, N = 7 from 5 mice; DES, N = 7 from 5 mice). Dark color is positive staining. (*D*) Violin plots of RNA expression of mesenchyme marker genes *Vim* and *Hoxa10* in PND5 control or DES-exposed epithelium or mesenchyme. (*E*) Ridge plots of partial EMT score for control and DES-exposed epithelial and mesenchymal cells. (*F*) Heatmap of expression of partial EMT genes, clustered by Euclidean distance, in both control and DES-exposed epithelium and mesenchyme. (*G*) Violin plots of RNA expression of the indicated EMT-controlling TFs, in control or DES-exposed epithelium or mesenchyme. Gene expression in all violin plots is significantly different unless marked as “ns.”

In addition to its transcriptional activity, β-catenin has an important role in supporting the cytoskeleton that is critical for integrity of epithelial monolayers. At bicellular adherens junctions, β-catenin bridges extracellular connections between cadherin molecules to the intracellular actin cytoskeleton through binding both the E-cadherin C-terminus and α-catenin (49). The increased active β-catenin in the epithelial cytoplasmic fraction suggested that these cytoskeletal features might change in response to DES. E-cadherin localized to epithelial cell–cell junctions in both groups, but in DES-exposed uteri, there was also basal plasma membrane staining (Fig. 3*A*). Furthermore, control PND5 uteri had a clearly delineated basal lamina that was difficult to visualize in DES-exposed uteri (Fig. 3*A*). Taken together with the expanded number of ligand–receptor pairs related to cell adhesion in the DES-exposed uterus (Fig. 2*A*), these findings suggest that at PND5 the two groups have differences in epithelial adhesion characteristics.

Bidirectional signaling between ECM molecules and epithelial cells is vital for the initial establishment and maintenance of apical–basal cell orientation and cell adhesion to the underlying basement membrane. Key mediators of this communication include ECM collagens and laminins. Many collagen genes were highly expressed in the PND5 uterus, particularly following DES exposure (*SI Appendix*, Fig. S4*B*). Laminin gene expression was also dysregulated following DES exposure, with strong upregulation of *Lama5* in the epithelium (*SI Appendix*, Fig. S6*A*). Adhesion of epithelial cells to the underlying basement membrane relies largely on the epithelial cell expression of the integrin heterodimers that serve as laminin receptors: integrins α3β1, α6β1, α7β1, and α6β4 (50). *Itgb1* was strongly expressed in both epithelial and mesenchymal cells regardless of treatment group, but *Itgb4* was only expressed at a low level following DES exposure, not in controls (*SI Appendix*, Fig. S6*B*). In control epithelial cells, *Itga6* was expressed at a very low level, but it was highly upregulated in epithelium following DES exposure; neither group expressed *Itga3* or *Itga7*. These findings suggest that DES epithelial cells likely expressed mainly integrins α6β1 and α6β4, appropriate laminin receptors to cause increased adhesiveness to the underlying basement membrane.

Epithelial sheets maintain their structure through stabilizing actomyosin cytoskeletal networks in a belt near the apical region (51). Developing intestinal epithelium has an additional actomyosin network at the basal region, except at regions where crypts will form by protrusion of the epithelium into the underlying mesenchyme (52). To determine if the changes we observed in collagen, laminin, and integrin expression were correlated with alterations in establishment of epithelial actomyosin skeletal networks in the uterus, we examined localization of filamentous actin. In control PND5 epithelial cells, actin staining was visible in a diffuse pattern at the apical region and in puncta at cell–cell junctions (Fig. 3*B*). In comparison, DES PND5 epithelium had similar puncta at cell–cell junctions but had strong actin staining at the basal region adjacent to the basement membrane and minimal apical actin. By PND9, controls had increased overall actin staining, with higher intensity staining at the apical region and some regions that had clear basal actin staining (Fig. 3*C*). In contrast, DES PND9 epithelial cells had distinct basal region actin staining and there was still not a clear apical actomyosin network as in controls. Despite the differences in apical actin, tight junction formation marked by ZO-1 staining was similar in both treatment groups (*SI Appendix*, Fig. S6*C*). The persistent alterations in both the apical and basal actomyosin networks indicate that alterations in the expression of ECM genes and their cellular receptors in response to DES exposure alters epithelial apical–basal polarity.

Formation of uterine glands occurs postnatally, beginning as invaginations of luminal epithelium into the mesenchyme (19). This type of morphogenetic process involves constriction of apical actomyosin networks to result in epithelial bending, often accompanied by basal actomyosin network relaxation and a partial EMT (17, 53–56). Indeed, mesenchymal markers *Vim* and *Hoxa10* were expressed in control epithelial cells (Fig. 3*D*), albeit at lower levels than in mesenchymal cells, consistent with the possibility that control epithelium was in a partial mesenchymal state. In the epithelium, both of these genes were reduced in DES compared to controls, with the greatest reduction observed for *Hoxa10*. To gain a better understanding of the potential EMT state of the epithelial cells, we utilized a partial EMT scoring system (57). Control epithelial cells had a higher partial EMT score than DES-exposed cells (Fig. 3*E*). A heat map of the expression of those partial EMT markers demonstrated that control and DES-exposed epithelium and control and DES-exposed mesenchyme expressed different subsets of these genes (Fig. 3*F* and *SI Appendix*, Fig. S6*D*). The decreased overall expression of partial EMT marker genes in the DES-exposed epithelium was also evident in the heatmap. We conclude that control epithelial cells retain a partial EMT program that is lost in DES-exposed epithelial cells.

Three TF families can control entry into EMT: Snail, Twist, and ZEB (58). Of these, only the *Zeb* genes were significantly expressed in PND5 uterine cells (Fig. 3*G* and Datasets S6 and S7). *Zeb1* and *Zeb2* were downregulated in DES-exposed epithelium, though still highly expressed in mesenchyme (Fig. 3*G* and *SI Appendix*, Fig. S6*E*). These findings suggest that DES-induced repression of *Zeb1* and *Zeb2* expression is responsible for the loss of the normal partial EMT that exists in unexposed epithelial cells.

### DES Exposure Leads to Increased Chromatin Accessibility at Enhancers.

To determine the mechanisms underlying the DES effects, we used our single nucleus multiomics data to link chromatin accessibility to RNA expression data from each cell. Using the annotation based on RNA expression, we subset epithelial and mesenchymal cells and examined their chromatin accessibility. Standard dimensional reduction and clustering was based on ATAC data from both epithelial cells and mesenchymal cells, with cells from control and DES-exposed mice integrated together. In epithelium, DES cells were generally nonoverlapping with control cells in the resulting UMAP (*SI Appendix*, Fig. S7 *A* and *B*). Although cells from both exposure groups were detected in all epithelial cell ATAC clusters, control cells were enriched in clusters 0 and 1 and DES cells were enriched in clusters 2 and 4 (*SI Appendix*, Fig. S7*C*). In epithelial cells, there were 5,515 peaks gained with DES exposure, and 653 lost (Dataset S11). In mesenchyme, the UMAP of cells based on ATAC data appeared more heterogenous than epithelial cells (*SI Appendix*, Fig. S7 *D* and *E*). However, control cells were enriched in clusters 1, 2, and 3 and DES cells were mainly in cluster 0 (*SI Appendix*, Fig. S7*F*). Only 606 peaks were gained with DES exposure, and 303 lost (Dataset S12).

**Fig. 4.**
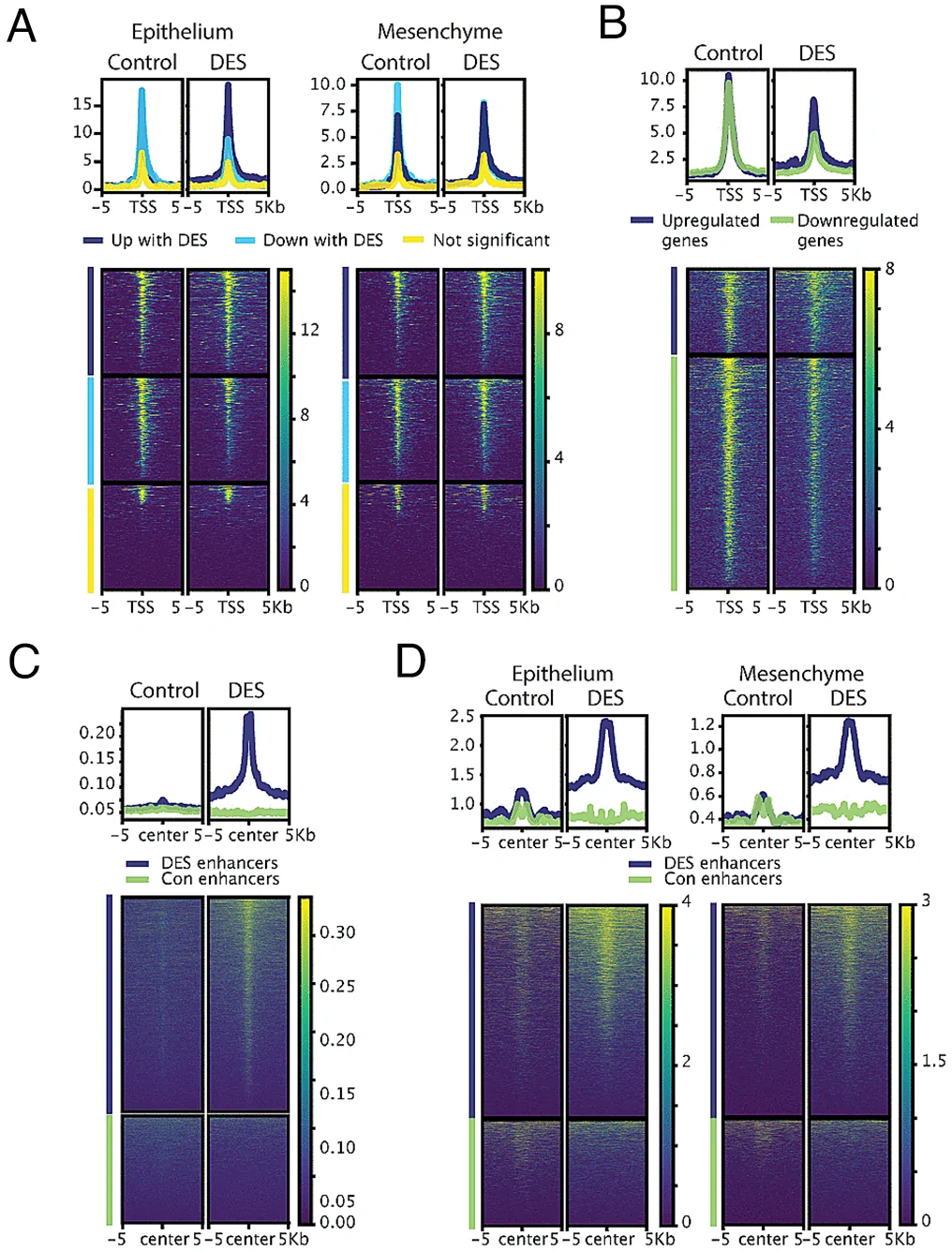
DES promotes chromatin accessibility and alters ERα binding, especially at differential enhancers. (*A*) Metaplots and heatmaps of pseudobulk ATAC signal at TSS of the top 300 differentially expressed (DES/Control) or 300 randomly selected nonsignificantly different genes from epithelium and mesenchyme. (*B*) Metaplots and heatmaps of whole uterine ERα ChIP reads at TSS of 660 upregulated and 1748 downregulated genes that were altered in the same direction in both epithelium and mesenchyme. See also *SI Appendix*, Fig. S7*G*. (*C*) Metaplots and heatmaps of whole uterine ERα ChIP reads at control- or DES-specific enhancers identified in (28). (*D*) Metaplots and heatmaps of pseudobulk ATAC signal from control or DES-exposed epithelium and mesenchyme at control- or DES-specific enhancers (28).

To assess the specificity of changes in accessibility in the two cell types with DES exposure, a pseudobulk, normalized ATAC read density was calculated at the transcription start site (TSS) of all detected genes, and sorted by differential expression status (upregulated by DES, downregulated by DES, or not significantly changed; Datasets S6 and S7). DES resulted in only minimal accessibility changes at the TSS regardless of cell type or differential expression (*SI Appendix*, Fig. S7*G*). Restriction to the top 300 up- and downregulated DEGs revealed decreased accessibility in downregulated DEGs in both epithelium and mesenchyme (Fig. 4*A*). The general disconnect between chromatin accessibility and gene expression in the case of DES-upregulated DEGs highlights the importance of coactivators, corepressors, or other regulatory mechanisms in controlling gene expression (24, 59–61).

DES effects are mediated by ERα, and therefore we wanted to know where ERα binds chromatin in uterine cells at PND5. To do so, we performed ERα ChIPseq on PND5 whole uteri from DES-exposed or unexposed mice. DES exposure resulted in 4,438 unique ERα peaks (of 5,037 total; Dataset S13), compared to only 1,285 peaks unique to controls (of 1,884 total; Dataset S14). Considering only the TSS of DEGs in DES vs control epithelial and mesenchymal cells (from the multiomics data), there was more ERα binding in controls than DES-exposed cells (Fig. 4*B*). Binding was decreased at the TSS of both up and downregulated DEGs that were shared between epithelium and mesenchyme (Fig. 4*B*); the same pattern was observed when DEGs were split by cell type (*SI Appendix*, Fig. S7*H*). We next examined ERα binding at enhancer locations that have differential H3K27ac marks following DES exposure at PND5 (28). There was a robust increase in ERα binding in DES-exposed uteri at DES-specific enhancers (Fig. 4*C*), consistent with previous observations that enhancer activation mediates DES effects on the PND5 uterus (28). In contrast, neither treatment group had ERα binding at control-specific enhancers (Fig. 4*C*). To determine how cell type–specific changes in accessibility correlated with the differential enhancers, pseudobulk ATAC read density was mapped at these sites. Accessible chromatin at DES-specific enhancers increased with DES, in both epithelium and mesenchyme, with DES-specific sites more accessible than control-specific sites (Fig. 4*D*). Control-specific enhancers had similarly low levels of ATAC signal in both exposure groups. Taken together, these data demonstrated increased chromatin accessibility co-occurring with ERα binding at DES-specific enhancer sites, but not promoter sites, in both epithelial and mesenchymal DES-exposed cells.

### DES-Induced Changes in Chromatin Accessibility at DEGs Correspond to Changes in ERα Binding Sites and Chromatin Looping.

To understand how DES changes the overall chromatin landscape to alter gene expression, we examined specific genes that had differential expression and differential accessibility within the same cell. This process enabled analysis of the correlation (referred to as linkage) between the presence or absence of nearby ATAC peaks and the expression of any gene. Additionally, to assess changes in chromatin architecture, we performed HiC-seq on whole uteri from DES-exposed or unexposed mice at PND5. Of 17,586 total loops found in DES-exposed uteri, and 15,220 in control uteri, 402 loops were gained and 267 lost with DES exposure (Dataset S15). Peaks from bulk ChIPseq of ERα binding sites and loops from HiC sequencing were overlaid with the aggregate single cell ATAC data and gene-specific linkages to create a picture of how changes in RNA expression could be tied to accessibility, chromatin looping, and ERα binding. Examples follow that illustrate where different combinations of chromatin interactions and ERα binding sites were present at the same locations as cell type–specific changes in ATAC peak strength and RNA expression.

**Fig. 5.**
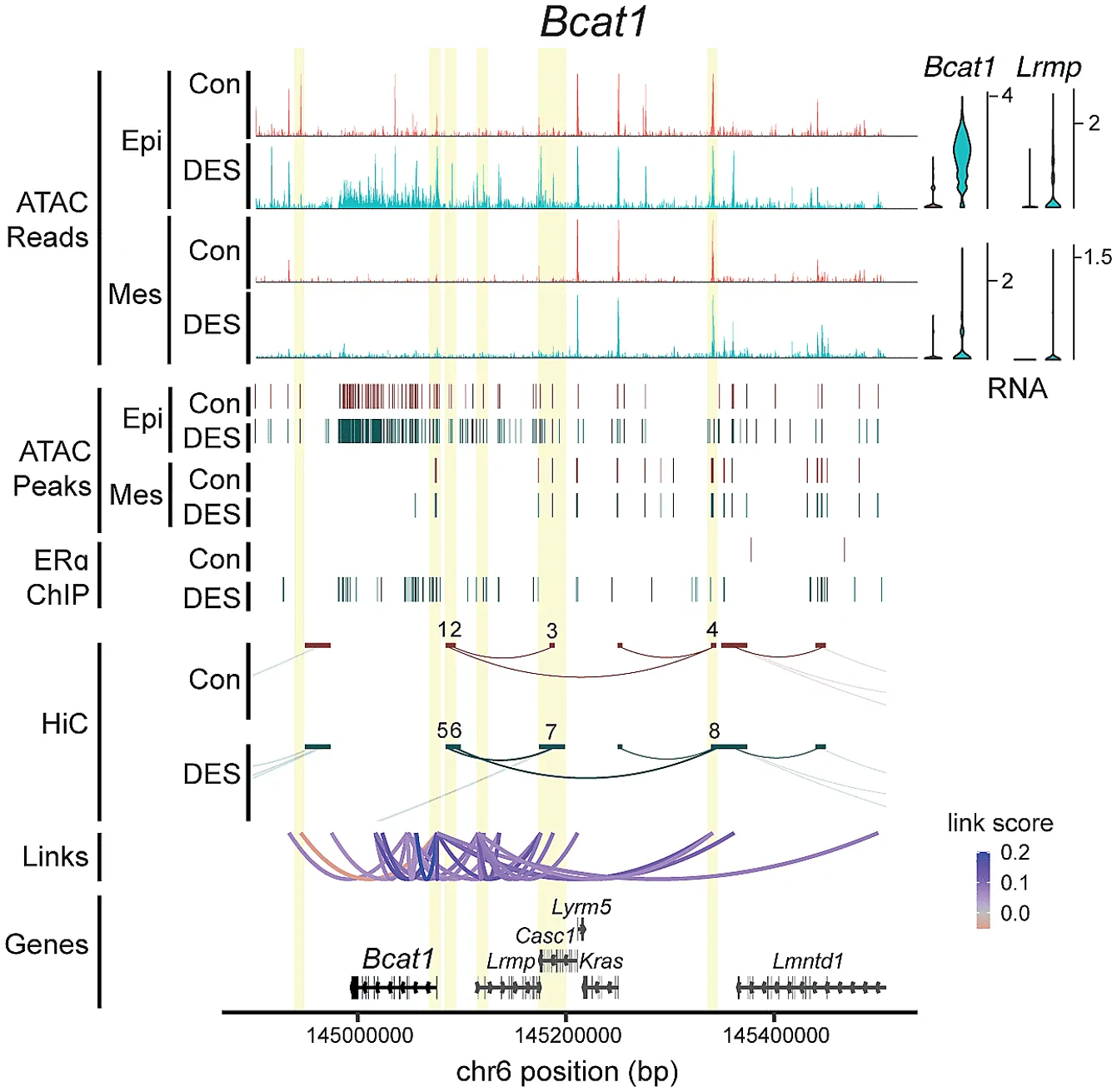
Alterations in chromatin accessibility, looping, and ERα binding co-occur at common sites in the DES-exposed uterine epithelium. Gene tracks at area surrounding *Bcat1* and *Lrmp*, including normalized pseudobulk ATAC signal and peaks in control or DES-exposed uterine epithelial or mesenchymal cells, ERα ChIP peaks from control or DES-exposed whole uterus, HiCseq loops from control or DES-exposed whole uterus, and linkage of RNA expression to open chromatin based on cell-specific ATAC signal. Yellow bars denote areas of interest mentioned in the main text. 1, control loop at 5’ of promoter region of *Bcat1*; 2, control loop at 3’ of promoter region of *Bcat1*; 3, control loop within *Casc1* with no ERα binding site; 4, control loop near *Lmntd1* with DES-specific ERα binding site; 5, DES loop at 5’ of promoter region of *Bcat1*; 6, DES loop at 3’ of promoter region of *Bcat1*; 7, expanded DES loop within *Casc1* with no ERα binding site; 8, DES loop near *Lmntd1* with DES-specific ERα binding site.

An example of changes in epithelium-specific gene expression linked to changes in chromatin accessibility, ERα binding sites and chromatin looping was provided by the estrogen-regulated gene *Bcat1* (Fig. 5). The *Bcat1* locus had extensive linkages across a ~400 kb region that included 5 additional genes; *Lrmp* was the closest and was also upregulated in DES epithelium. Almost all these linkages were positively associated with gene expression and were located at numerous ERα binding sites at open chromatin that was unique to the DES sample. Altered loop structure was observed between the *Bcat1* promoter region and a site that had increased ATAC signal in control epithelial cells, suggesting a potential repressor site. There was a very interesting change in chromatin looping between the *Bcat1* promoter and two upstream regions (−80 Kb and −275 Kb). In controls, the 5′ end of this promoter region loop was connected to a site adjacent to *Lmntd1* that contained an ERα binding site, and the 3′ end of this promoter region loop was looped to a site within the *Casc1* gene with no ERα binding site. In DES cells, the *Bcat1* promoter region loop was switched, with the 5′ end connected to *Casc1* and the 3′ end connected to the site adjacent to *Lmntd1* but extended to include an ERα binding site in accessible chromatin in epithelial cells. This chromatin structure rearrangement likely brought an active enhancer region to *Bcat1* that highly upregulated this gene.

Mesenchyme had fewer DEGs near differentially accessible sites. One area of interest was the chromosome 7 locus that contains *Prss23* and *Me3*, which had extensive linkages positively correlated with upregulation by DES in the mesenchyme. At the *Me3* promoter, there was a DES-specific increase in chromatin accessibility in mesenchyme that overlapped a DES-specific chromatin loop and ERα binding sites (*SI Appendix*, Fig. S8). This loop was linked to a second DES-specific chromatin loop at the *Prss23* promoter that had an ERα binding site and accessible chromatin; accessible chromatin was also present in epithelial cells. A third DES-specific chromatin loop was upstream of the nearby *Picalm* gene at a location with an ERα binding site and minimally increased ATAC signal in DES mesenchyme. This loop was connected directly to the *Picalm* promoter and indirectly to both the *Prss23* and *Me3* promoters. For these genes, expression appeared to be tied to new chromatin looping sites driven by DES-induced ERα binding rather than large changes in chromatin accessibility at the loop sites.

Together, these data support the concept that alterations in cell type–specific gene expression are induced by the combined impacts of changes in chromatin accessibility, chromatin looping, ERα binding, and the association of coactivators and corepressors. The specific mechanisms utilized differ across distinct regions of the genome, but all depend on the presence of ERα in the relevant cell type.

## Discussion

**Fig. 6.**
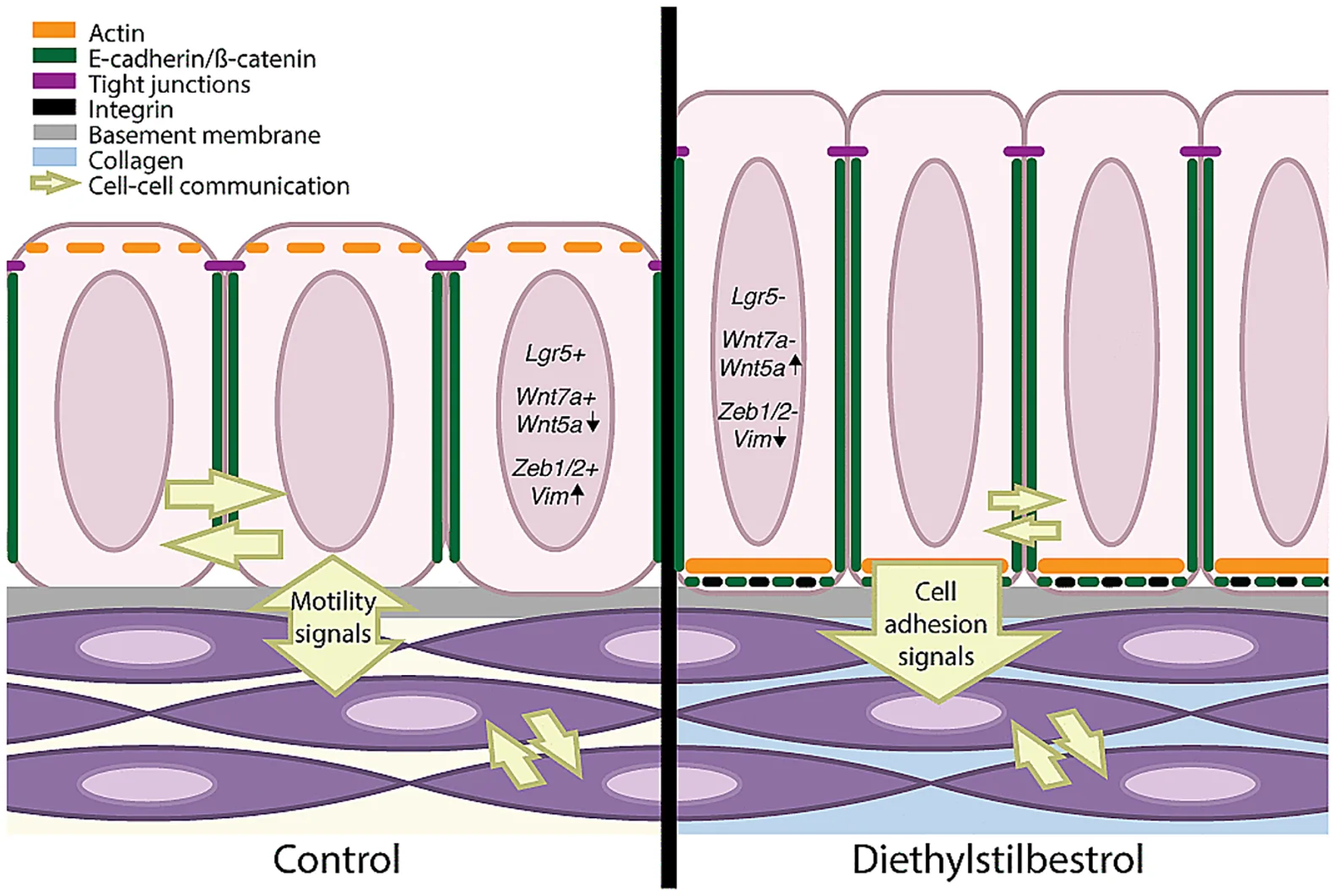
Neonatal DES exposure results in changes to the epithelial stem cell population, cell–cell communication, Wnt signaling, apical–basal polarity, and EMT status. Model of control and DES-exposed uterine epithelium and mesenchyme. Genes in the nucleus of epithelial cells reflect key expression differences in response to DES exposure. Yellow arrows indicate direction, amount, and nature of cell–cell signaling. Orange lines within epithelial cells reflect change in actin localization from apical to basal. Green lines represent similar expression of E-cadherin & β-catenin laterally, with aberrant expression punctate in the basal region of the DES-exposed epithelium. Black dots reflect increased integrin expression in the basal region of the DES-exposed epithelium. Increased collagen expression in DES mesenchyme shown in blue.

Here we found that developmental exposure to DES promotes premature expression of ERα in uterine epithelium, activates uterine epithelial Wnt signaling, and eliminates the *Lgr5*+ epithelial stem cell population. Intercellular communication is dramatically reorganized, and the developing uterine epithelium loses its normal partial EMT character. Epithelial cell apical–basal polarity changes that under normal conditions likely support glandular development are also disrupted. At enhancers, DES exposure increases chromatin accessibility through promoting binding of ERα and causes cell type–specific changes in gene expression, most prominently in epithelial cells. At least some of the critical gene expression changes can be explained by cell type–specific changes in chromatin accessibility and chromatin looping at enhancer regions in concert with alterations in ERα binding. These findings suggest that ERα-mediated reprogramming of cell differentiation trajectories underlies the long-term phenotypic effects of developmental exposure to DES, and perhaps other estrogenic endocrine disrupting chemicals (Fig. 6).

Wnt/β-catenin signaling is a critical mediator of female reproductive tract development based on data from multiple animals including humans (62–65). Estrogens can both activate and inhibit Wnt signaling depending on the cell and tissue context. For example, ERα and β-catenin physically interact to promote transcription of target genes in colon and breast cancer cells, and estradiol promotes translocation of β-catenin to the nucleus in uterine stromal cells (66, 67). Estrogen can also act through a nongenomic mechanism to promote expression of Wnt ligands and frizzled receptors important for uterine epithelial growth (68). In the developing uterine epithelium it is clear based on the results presented here that the normal developmental balance in Wnt signaling is shifted to full activation by DES exposure. In contrast, in a mouse hepatocarcinoma model in which mice are treated with an ERα agonist, Wnt/β-catenin signaling is suppressed and there is a lower tumor burden and extended survival (69). What causes these differences in the downstream consequences of estrogenic chemical action on Wnt signaling is unknown, but the overall chromatin state and relative expression of coactivators/corepressors in the developing uterus and cancer cells may be key factors.

In addition to its important roles in development, LGR5 is a marker of adult epithelial stem cells in numerous tissues and of cancer stem cells (70). The endometrial epithelium is no exception, as LGR5 is required for both in vivo Wnt signaling-dependent endometrial gland development and ex vivo endometrial epithelial cell self-renewal in the context of organoids (20). Here, we extended the previous observation that estradiol treatment inhibits *Lgr5* gene expression in adult uterine tissue (21) to show that DES similarly inhibits *Lgr5* expression in the context of the neonatal, differentiating uterus. This finding likely explains the reduction in uterine gland development observed in adult mice exposed neonatally to either DES or the phytoestrogen genistein (14, 38). However, the presence of some glands in exposed uteri suggests that *Lgr5* expression must resume sufficiently to support their development, even if this does not occur by PND9 (Fig. 1*H*). Alternatively, an additional bipotential epithelial stem cell population could support the limited glandular development (71). Because LGR5 is suppressed by estrogenic chemicals in both developmental and adult settings, long term negative impacts of estrogenic endocrine disruption on stem cells and adult tissue functions could be explained by this mechanism. Furthermore, it raises the possibility that targeting LGR5 using hormonal manipulations could be a useful adjunct to other LGR5-targeting cancer therapies (70).

A partial EMT state is characteristic of epithelia in multiple organs during prenatal organogenesis and of neoplastic cells undergoing invasion or metastasis (72, 73). We uncovered a similar profile of partial EMT in control developing uterine epithelium, suggesting a need for uterine epithelial cells to have mesenchymal traits at this developmental stage in preparation for endometrial gland budding and extension into the stroma. It is likely that this partial EMT state is a result of incomplete epithelial morphogenesis of the Müllerian duct, which arises from mesoepithelial cells during prenatal development (74). During morphogenesis of mammary gland branching ducts, the leading epithelial cells undergo partial EMT so that they lose stable epithelial phenotypes and can invade the surrounding stroma (75). This is a tightly regulated process, influenced by Wnt signaling, growth factor signaling, and classical EMT TFs such as Snail, Twist, and Zeb family proteins. It is accompanied by expression of matrix metalloproteases that break down ECM material to facilitate epithelial migration into the stroma (76). Here, we show that similar mechanisms are likely operating in normal developing endometrium, where the epithelium expresses *Zeb1* and *Zeb2* and the epithelium and stroma express several matrix metalloproteases. DES exposure pushes the normal partial EMT toward a full epithelial state with stable adhesion to the basement membrane supported by a basal actin network, combined with reduced MMP expression and elevated stromal collagens. The combined impact of these changes likely underlies the observed impairment in uterine adenogenesis.

During prenatal female reproductive tract development from the Müllerian duct, cell–cell communication is vital to control the developmental plasticity of the tissue (77). This remains true during the mouse neonatal period when mesenchymal signals control epithelial differentiation patterns (31). The main ways this communication is regulated are through Wnt signaling and Hox TF-mediated gene expression (77). As reproductive maturity is reached, mesenchymal retinoic acid signaling becomes important for preventing endogenous estrogen-induced transdifferentiation of uterine epithelial cells into stratified squamous epithelium with basal cell differentiation (44). This mechanism, which is ERα-dependent, suggests a need for ongoing mesenchymal–epithelial communication to maintain proper epithelial identity. Restructuring of cell–cell communication following neonatal DES exposure (Fig. 2*A*), compounded by dramatic changes in mesenchyme ECM composition (Fig. 2*B* and *SI Appendix*, Fig. S4 *B* and *C*), clearly impairs maintenance of the normal epithelial phenotype and likely explains the basal cell metaplasia and squamous metaplasia observed in adult uterine epithelium of mice exposed neonatally to DES or genistein (16).

An important question regarding this work is why the developmental timing is so important in the disruptive effects of neonatal DES exposure. We propose that the critical factor is that DES (or other estrogenic chemicals) induces the expression and activation of ERα in epithelial cells when it is not normally present at sufficient levels to be functional (32), resulting in a loss of the partial EMT characteristics that are required for adenogenesis. The change in ERα functional status causes the epithelial cells to be susceptible to ERα-mediated differentiation signals that are activated in the presence of continued DES exposure over several days, at time points in which the protective effects of retinoic acid signaling are not yet active. These ERα-mediated signals promote global changes in chromatin accessibility and three-dimensional chromatin structure. Furthermore, the DES exposure leads to ECM deposition responses in the mesenchyme that impair cell–cell communication—likely also preventing quick reversal of the abnormal epithelial differentiation phenotypes when DES is discontinued. Later in development, processes such as retinoic acid signaling limit epithelial remodeling by either endogenous or exogenous estrogenic chemicals, protecting the uterine epithelium from long term phenotypic consequences.

During human reproductive tract development, there is no *Esr1* expression in Mullerian duct derivatives until >10 postconceptional weeks, and likely no presence of functional ERα signaling in the epithelium until sometime after 17 postconceptional weeks (64). However, in human endometrial organoids derived from 12 and 17 postconceptional week tissues, estrogenic endocrine disrupting compounds induce *ESR1* and *PGR* expression (64). If our proposal that the timing of ERα expression in the developing female reproductive tract is key for understanding the window of susceptibility to estrogenic endocrine disruptors, these findings suggest that the human female fetus is susceptible at least well into the second trimester. The well documented impact of DES exposure during fetal development on adult female reproductive tract differentiation and function validates this concept (2). Given that uterine adenogenesis is ongoing from the second trimester through puberty (3, 4), this susceptibility may persist. Indeed, neonatal exposure to soy based infant formula, a plant source of estrogenic chemical exposure, increases the incidence and/or severity of common adult human reproductive tract pathophysiological conditions, including uterine fibroids, heavy menstrual bleeding, and endometriosis (5, 6, 78, 79). Such pathologies could have their beginnings in the impact of environmental exposures on cell differentiation trajectories during development.

## Materials and Methods

Animal procedures were approved by the National Institute of Environmental Health Sciences (NIEHS) animal care and use committee under protocol 2007-038. Animal care, treatments and tissue collection were performed as described in (15). Tissues were fixed as described in (80). Single-nucleus isolation and sequencing protocol was adapted from (15). ChIP and HiC protocols were adapted from (24). Full details regarding analyses and other protocols can be found in Supplemental Methods, with key reagents and software described in a Key Reagent table. Code used for analysis is found at https://github.com/DrBrainbridge/PND5-snMultiomics.

## Supplementary Material

Appendix 01 (PDF)

Dataset S01 (CSV)

Dataset S02 (CSV)

Dataset S03 (CSV)

Dataset S04 (CSV)

Dataset S05 (CSV)

Dataset S06 (CSV)

Dataset S07 (CSV)

Dataset S08 (CSV)

Dataset S09 (CSV)

Dataset S10 (CSV)

Dataset S11 (CSV)

Dataset S12 (CSV)

Dataset S13 (CSV)

Dataset S14 (CSV)

Dataset S15 (CSV)

## Data Availability

Single nucleus multiomic sequencing (RNA & ATAC), ChIP-seq, and HiC-seq data have been deposited in NCBI BioProject (PRJNA1259040, PRJNA1259522, PRJNA1259523).

## References

[1] S. M. Ho , Environmental factors, epigenetics, and developmental origin of reproductive disorders. Reprod. Toxicol. **68**, 85–104 (2017). 27421580 10.1016/j.reprotox.2016.07.011 PMC5233640

[2] C. E. Reed, S. E. Fenton, Exposure to diethylstilbestrol during sensitive life stages: A legacy of heritable health effects. Birth Defects Res. Part C: Embryo Today: Rev. **99**, 134–146 (2013). 10.1002/bdrc.21035 PMC3817964 23897597

[3] T. E. Spencer, K. Hayashi, J. Hu, K. D. Carpenter, “Comparative developmental biology of the mammalian uterus” in Cur Top. Dev. Biol. (2005), vol. **68**, pp. 85–122. 10.1016/S0070-2153(05)68004-0 16124997

[4] M. A. Valdes-Dapena, The development of the uterus in late fetal life, infancy, and childhood. The Uterus **14**, 40–67 (1973).

[5] K. Upson, Q. E. Harmon, D. D. Baird, Soy-based infant formula feeding and ultrasound-detected uterine fibroids among young African-American women with no prior clinical diagnosis of fibroids. Environ. Health Perspect. **124**, 769–775 (2016). 26565393 10.1289/ehp.1510082 PMC4892927

[6] K. Upson, S. Sathyanarayana, D. Scholes, V. L. Holt, Early-life factors and endometriosis risk. Fertil. Steril. **104**, 964–971.e965 (2015). 26211883 10.1016/j.fertnstert.2015.06.040 PMC5328429

[7] W. N. Jefferson , Neonatal exposure to genistein disrupts ability of female mouse reproductive tract to support preimplantation embryo development and implantation. Biol. Reprod. **80**, 425–431 (2009). 19005167 10.1095/biolreprod.108.073171 PMC2677916

[8] W. N. Jefferson, E. Padilla-Banks, R. R. Newbold, Adverse effects on female development and reproduction in CD-1 mice following neonatal exposure to the phytoestrogen genistein at environmentally relevant doses. Biol. Reprod. **73**, 798–806 (2005). 15930323 10.1095/biolreprod.105.041277

[9] R. R. Newbold, E. P. Banks, B. Bullock, W. N. Jefferson, Uterine adenocarcinoma in mice treated neonatally with genistein. Cancer Res. **61**, 4325–4328 (2001). 11389053

[10] R. R. Newbold, B. C. Bullock, J. A. McLachlan, Uterine adenocarcinoma in mice following developmental treatment with estrogens: A model for hormonal carcinogenesis. Cancer Res. **50**, 7677–7681 (1990). 2174729

[11] F. Ran, S. T. Chen, M. Y. Li, D. D. Jin, Z. M. Yang, Effect of diethylstilbestrol on implantation and decidualization in mice. Int. J. Mol. Sci. **26**, 4122 (2025). 40362361 10.3390/ijms26094122 PMC12071748

[12] K. Hayashi , WNTs in the neonatal mouse uterus: Potential regulation of endometrial gland development. Biol. Reprod. **84**, 308–319 (2011). 20962251 10.1095/biolreprod.110.088161 PMC3071266

[13] W. W. Huang , Developmental diethylstilbestrol exposure alters genetic pathways of uterine cytodifferentiation. Mol. Endocrinol. **19**, 669–682 (2005). 15591538 10.1210/me.2004-0155

[14] W. N. Jefferson , Uterine patterning, endometrial gland development, and implantation failure in mice exposed neonatally to genistein. Environ. Health Perspect. **128**, 37001 (2020). 32186404 10.1289/EHP6336 PMC7138129

[15] E. Padilla-Banks , Developmental estrogen exposure in mice disrupts uterine epithelial cell differentiation and causes adenocarcinoma via Wnt/beta-catenin and PI3K/AKT signaling. PLoS Biol. **21**, e3002334 (2023). 37856394 10.1371/journal.pbio.3002334 PMC10586657

[16] A. A. Suen, W. N. Jefferson, C. J. Williams, C. E. Wood, Differentiation patterns of uterine carcinomas and precursor lesions induced by neonatal estrogen exposure in mice. Toxicol. Pathol. **46**, 574–596 (2018). 29895210 10.1177/0192623318779326 PMC6027618

[17] C. X. Zhang, R. Y. Huang, G. Sheng, J. P. Thiery, Epithelial-mesenchymal transition. Cell **188**, 5436–5486 (2025). 41043405 10.1016/j.cell.2025.08.033

[18] M. A. Nieto, R. Y. Huang, R. A. Jackson, J. P. Thiery, EMT: 2016. Cell **166**, 21–45 (2016). 27368099 10.1016/j.cell.2016.06.028

[19] P. S. Cooke, T. E. Spencer, F. F. Bartol, K. Hayashi, Uterine glands: Development, function and experimental model systems. Mol. Hum. Reprod. **19**, 547–558 (2013). 23619340 10.1093/molehr/gat031 PMC3749806

[20] R. Seishima , Neonatal Wnt-dependent Lgr5 positive stem cells are essential for uterine gland development. Nat. Commun. **10**, 5378 (2019). 31772170 10.1038/s41467-019-13363-3 PMC6879518

[21] X. Sun, L. Jackson, S. K. Dey, T. Daikoku, In pursuit of leucine-rich repeat-containing G protein-coupled receptor-5 regulation and function in the uterus. Endocrinology **150**, 5065–5073 (2009). 19797400 10.1210/en.2009-0690 PMC2775985

[22] S. C. Hewitt, K. S. Korach, Estrogen receptors: New directions in the new millennium. Endocr. Rev. **39**, 664–675 (2018). 29901737 10.1210/er.2018-00087 PMC6173474

[23] S. C. Hewitt , Research resource: Whole-genome estrogen receptor alpha binding in mouse uterine tissue revealed by ChIP-seq. Mol. Endocrinol. **26**, 887–898 (2012). 22446102 10.1210/me.2011-1311 PMC3355558

[24] W. N. Jefferson, T. Wang, E. Padilla-Banks, C. J. Williams, Unexpected nuclear hormone receptor and chromatin dynamics regulate estrous cycle dependent gene expression. Nucleic Acids Res. **52**, 10897–10917 (2024). 39166489 10.1093/nar/gkae714 PMC11472041

[25] M. Madhavan, R. Arora, Tracing early postnatal lineage of progesterone receptor in the mouse uterus. Mol. Reprod. Dev. **89**, 69–69 (2022).

[26] S. Jia, F. Zhao, Single-cell transcriptomic profiling of the neonatal oviduct and uterus reveals new insights into upper Mullerian duct regionalization. FASEB J. **38**, e23632 (2024). 38686936 10.1096/fj.202400303R PMC11095678

[27] Y. Hao , Dictionary learning for integrative, multimodal and scalable single-cell analysis. Nat. Biotechnol. **42**, 293–304 (2024). 37231261 10.1038/s41587-023-01767-y PMC10928517

[28] W. N. Jefferson , Widespread enhancer activation via ERalpha mediates estrogen response in vivo during uterine development. Nucleic Acids Res. **46**, 5487–5503 (2018). 29648668 10.1093/nar/gky260 PMC6009594

[29] W. Winuthayanon, S. C. Hewitt, K. S. Korach, Uterine epithelial cell estrogen receptor alpha-dependent and -independent genomic profiles that underlie estrogen responses in mice. Biol. Reprod. **91**, 110 (2014). 25210133 10.1095/biolreprod.114.120170 PMC4434925

[30] T. E. Spencer, M. T. Lowke, K. M. Davenport, P. Dhakal, A. M. Kelleher, Single-cell insights into epithelial morphogenesis in the neonatal mouse uterus. Proc. Natl. Acad. Sci. U.S.A. **120**, e2316410120 (2023). 38019863 10.1073/pnas.2316410120 PMC10710066

[31] T. Kurita, P. S. Cooke, G. R. Cunha, Epithelial-stromal tissue interaction in paramesonephric (Mullerian) epithelial differentiation. Dev. Biol. **240**, 194–211 (2001). 11784056 10.1006/dbio.2001.0458

[32] S. Yamashita, R. R. Newbold, J. A. McLachlan, K. S. Korach, Developmental pattern of estrogen receptor expression in female mouse genital tracts. Endocrinology **125**, 2888–2896 (1989). 2583044 10.1210/endo-125-6-2888

[33] E. L. Boutin, E. Battle, G. R. Cunha, The response of female urogenital tract epithelia to mesenchymal inductors is restricted by the germ layer origin of the epithelium: Prostatic inductions. Differentiation **48**, 99–105 (1991). 1773919 10.1111/j.1432-0436.1991.tb00248.x

[34] G. R. Cunha, Stromal induction and specification of morphogenesis and cytodifferentiation of the epithelia of the Mullerian ducts and urogenital sinus during development of the uterus and vagina in mice. J. Exp. Zool. **196**, 361–370 (1976). 932664 10.1002/jez.1401960310

[35] D. Dimitrov , Comparison of methods and resources for cell-cell communication inference from single-cell RNA-Seq data. Nat. Commun. **13**, 3224 (2022). 35680885 10.1038/s41467-022-30755-0 PMC9184522

[36] D. Bagnard , Semaphorin 3A-vascular endothelial growth factor-165 balance mediates migration and apoptosis of neural progenitor cells by the recruitment of shared receptor. J. Neurosci. **21**, 3332–3341 (2001). 11331362 10.1523/JNEUROSCI.21-10-03332.2001 PMC6762465

[37] J. A. Rizo , Uterine organoids reveal insights into epithelial specification and plasticity in development and disease. Proc. Natl. Acad. Sci. U.S.A. **122**, e2422694122 (2025). 39883834 10.1073/pnas.2422694122 PMC11804710

[38] A. Yoshida, R. R. Newbold, D. Dixon, Effects of neonatal diethylstilbestrol (DES) exposure on morphology and growth patterns of endometrial epithelial cells in CD-1 mice. Toxicol. Pathol. **27**, 325–333 (1999). 10356709 10.1177/019262339902700308

[39] X. M. Meng, D. J. Nikolic-Paterson, H. Y. Lan, TGF-beta: The master regulator of fibrosis. Nat. Rev. Nephrol. **12**, 325–338 (2016). 27108839 10.1038/nrneph.2016.48

[40] R. R. Newbold, W. N. Jefferson, E. Padilla-Banks, Long-term adverse effects of neonatal exposure to bisphenol A on the murine female reproductive tract. Reprod. Toxicol. **24**, 253–258 (2007). 17804194 10.1016/j.reprotox.2007.07.006 PMC2043380

[41] T. Y. Feinberg, R. G. Rowe, T. L. Saunders, S. J. Weiss, Functional roles of MMP14 and MMP15 in early postnatal mammary gland development. Development **143**, 3956–3968 (2016). 27633994 10.1242/dev.136259 PMC5117140

[42] J. Tan , Stromal matrix metalloproteinase-11 is involved in the mammary gland postnatal development. Oncogene **33**, 4050–4059 (2014). 24141782 10.1038/onc.2013.434

[43] H. E. Zhou, X. Zhang, W. B. Nothnick, Disruption of the TIMP-1 gene product is associated with accelerated endometrial gland formation during early postnatal uterine development. Biol. Reprod. **71**, 534–539 (2004). 15084483 10.1095/biolreprod.104.029181

[44] Y. Yin , Retinoic acid antagonizes estrogen signaling to maintain adult uterine cell fate. Proc. Natl. Acad. Sci. U.S.A. **122**, e2416089122 (2025). 39874292 10.1073/pnas.2416089122 PMC11804538

[45] K. Qin , Canonical and noncanonical Wnt signaling: Multilayered mediators, signaling mechanisms and major signaling crosstalk. Genes Dis. **11**, 103–134 (2024). 37588235 10.1016/j.gendis.2023.01.030 PMC10425814

[46] A. Wodarz, R. Nusse, Mechanisms of Wnt signaling in development. Annu. Rev. Cell Dev. Biol. **14**, 59–88 (1998). 9891778 10.1146/annurev.cellbio.14.1.59

[47] B. Sen , Mechanically induced nuclear shuttling of beta-catenin requires co-transfer of actin. Stem Cells **40**, 423–434 (2022). 35278073 10.1093/stmcls/sxac006 PMC9633329

[48] S. Yamazaki, K. Yamamoto, P. de Lanerolle, M. Harata, Nuclear F-actin enhances the transcriptional activity of beta-catenin by increasing its nuclear localization and binding to chromatin. Histochem Cell Biol. **145**, 389–399 (2016). 26900020 10.1007/s00418-016-1416-9

[49] W. J. Nelson, R. Nusse, Convergence of Wnt, beta-catenin, and cadherin pathways. Science **303**, 1483–1487 (2004). 15001769 10.1126/science.1094291 PMC3372896

[50] W. Xuan, Y. Yao, Laminin receptors in peripheral tissues: Functions revealed by analysis of knockout mice. Am. J. Pathol. **195**, 2303–2319 (2025). 40945909 10.1016/j.ajpath.2025.08.007 PMC12799409

[51] M. A. Pickett, V. F. Naturale, J. L. Feldman, A polarizing issue: Diversity in the mechanisms underlying apico-basolateral polarization in vivo. Annu. Rev. Cell Dev. Biol. **35**, 285–308 (2019). 31461314 10.1146/annurev-cellbio-100818-125134

[52] A. Barai , A multicellular star-shaped actin network underpins epithelial organization and connectivity. Nat. Commun. **16**, 6201 (2025). 40615382 10.1038/s41467-025-61438-1 PMC12227619

[53] M. Gracia , Mechanical impact of epithelial-mesenchymal transition on epithelial morphogenesis in *Drosophila*. Nat. Commun. **10**, 2951 (2019). 31273212 10.1038/s41467-019-10720-0 PMC6609679

[54] D. Krueger, P. Tardivo, C. Nguyen, S. De Renzis, Downregulation of basal myosin-II is required for cell shape changes and tissue invagination. EMBO J. **37**, e100170 (2018). 30442834 10.15252/embj.2018100170 PMC6276876

[55] A. C. Martin, M. Kaschube, E. F. Wieschaus, Pulsed contractions of an actin-myosin network drive apical constriction. Nature **457**, 495–499 (2009). 19029882 10.1038/nature07522 PMC2822715

[56] Y. Wang, D. Stonehouse-Smith, M. T. Cobourne, J. B. A. Green, M. Seppala, Cellular mechanisms of reverse epithelial curvature in tissue morphogenesis. Front. Cell. Dev. Biol. **10**, 1066399 (2022). 36518538 10.3389/fcell.2022.1066399 PMC9742543

[57] S. V. Puram , Single-cell transcriptomic analysis of primary and metastatic tumor ecosystems in head and neck cancer. Cell **171**, 1611–1624.e1624 (2017). 29198524 10.1016/j.cell.2017.10.044 PMC5878932

[58] J. Xu, S. Lamouille, R. Derynck, TGF-beta-induced epithelial to mesenchymal transition. Cell Res. **19**, 156–172 (2009). 19153598 10.1038/cr.2009.5 PMC4720263

[59] C. Y. Lin , Whole-genome cartography of estrogen receptor alpha binding sites. PLoS Genet. **3**, e87 (2007). 17542648 10.1371/journal.pgen.0030087 PMC1885282

[60] H. U. Osmanbeyoglu , Estrogen represses gene expression through reconfiguring chromatin structures. Nucleic Acids Res. **41**, 8061–8071 (2013). 23821662 10.1093/nar/gkt586 PMC3783169

[61] F. Stossi, Z. Madak-Erdogan, B. S. Katzenellenbogen, Estrogen receptor alpha represses transcription of early target genes via p300 and CtBP1. Mol. Cell. Biol. **29**, 1749–1759 (2009). 19188451 10.1128/MCB.01476-08 PMC2655624

[62] L. Fontana, B. Gentilin, L. Fedele, C. Gervasini, M. Miozzo, Genetics of Mayer-Rokitansky-Kuster-Hauser (MRKH) syndrome. Clin. Genet. **91**, 233–246 (2017). 27716927 10.1111/cge.12883

[63] A. Kobayashi, R. R. Behringer, Developmental genetics of the female reproductive tract in mammals. Nat. Rev. Genet. **4**, 969–980 (2003). 14631357 10.1038/nrg1225

[64] V. Lorenzi , Spatiotemporal cellular map of the developing human reproductive tract. Nature **650**, 428–437 (2026). 41407855 10.1038/s41586-025-09875-2 PMC12893920

[65] P. Philibert , Identification and functional analysis of a new WNT4 gene mutation among 28 adolescent girls with primary amenorrhea and mullerian duct abnormalities: A French collaborative study. J. Clin. Endocrinol. Metab. **93**, 895–900 (2008). 18182450 10.1210/jc.2007-2023

[66] A. P. Kouzmenko , Wnt/beta-catenin and estrogen signaling converge in vivo. J. Biol. Chem. **279**, 40255–40258 (2004). 15304487 10.1074/jbc.C400331200

[67] V. Rider , Progesterone initiates Wnt-beta-catenin signaling but estradiol is required for nuclear activation and synchronous proliferation of rat uterine stromal cells. J. Endocrinol. **191**, 537–548 (2006). 17170212 10.1677/joe.1.07030

[68] X. Hou, Y. Tan, M. Li, S. K. Dey, S. K. Das, Canonical Wnt signaling is critical to estrogen-mediated uterine growth. Mol. Endocrinol. **18**, 3035–3049 (2004). 15358837 10.1210/me.2004-0259 PMC4280566

[69] M. Bhat , Estrogen receptor 1 inhibition of Wnt/beta-catenin signaling contributes to sex differences in hepatocarcinogenesis. Front. Oncol. **11**, 777834 (2021). 34881186 10.3389/fonc.2021.777834 PMC8645636

[70] W. Wang, N. A. Lokman, S. C. Barry, M. K. Oehler, C. Ricciardelli, LGR5: An emerging therapeutic target for cancer metastasis and chemotherapy resistance. Cancer Metastasis Rev. **44**, 23 (2025). 39821694 10.1007/s10555-024-10239-x PMC11742290

[71] S. Jin, Bipotent stem cells support the cyclical regeneration of endometrial epithelium of the murine uterus. Proc. Natl. Acad. Sci. U.S.A. **116**, 6848–6857 (2019). 30872480 10.1073/pnas.1814597116 PMC6452687

[72] J. Dong , Single-cell RNA-seq analysis unveils a prevalent epithelial/mesenchymal hybrid state during mouse organogenesis. Genome Biol. **19**, 31 (2018). 29540203 10.1186/s13059-018-1416-2 PMC5853091

[73] A. Dongre, R. A. Weinberg, New insights into the mechanisms of epithelial-mesenchymal transition and implications for cancer. Nat. Rev. Mol. Cell Biol. **20**, 69–84 (2019). 30459476 10.1038/s41580-018-0080-4

[74] G. D. Orvis, R. R. Behringer, Cellular mechanisms of Mullerian duct formation in the mouse. Dev. Biol. **306**, 493–504 (2007). 17467685 10.1016/j.ydbio.2007.03.027 PMC2730733

[75] S. Ingthorsson, G. A. Traustadottir, T. Gudjonsson, Breast morphogenesis: From normal development to cancer. Adv. Exp. Med. Biol. **1464**, 29–44 (2025). 39821019 10.1007/978-3-031-70875-6_3

[76] T. Y. Feinberg , Divergent matrix-remodeling strategies distinguish developmental from neoplastic mammary epithelial cell invasion programs. Dev. Cell **47**, 145–160 (2018). 30269950 10.1016/j.devcel.2018.08.025 PMC6317358

[77] F. Zhao, H. H. Yao, A tale of two tracts: History, current advances, and future directions of research on sexual differentiation of reproductive tractsdagger. Biol. Reprod. **101**, 602–616 (2019). 31058957 10.1093/biolre/ioz079 PMC6791057

[78] C. R. Langton, Q. E. Harmon, K. Upson, D. D. Baird, Soy-based infant formula feeding and uterine fibroid development in a prospective ultrasound study of Black/African-American women. Environ. Health Perspect. **131**, 17006 (2023). 36696103 10.1289/EHP11089 PMC9875846

[79] K. Upson, Q. E. Harmon, S. K. Laughlin-Tommaso, D. M. Umbach, D. D. Baird, Soy-based infant formula feeding and heavy menstrual bleeding among young African American women. Epidemiology **27**, 716–725 (2016). 27196806 10.1097/EDE.0000000000000508 PMC5425950

[80] L. Jimenez-Gracia , FixNCut: Single-cell genomics through reversible tissue fixation and dissociation. Genome Biol. **25**, 81 (2024). 38553769 10.1186/s13059-024-03219-5 PMC10979608

