# Supplementary material for: Neonatal diethylstilbestrol exposure disrupts uterine epithelial apical–basal polarity and partial EMT state
Source: Proc Natl Acad Sci U S A. 2026 Aug 17;123(34):e2603458123. doi: 10.1073/pnas.2603458123 (PMC13506072; doi:10.1073/pnas.2603458123)
Supplement: Supplementary file 1 [file pnas.2603458123.sapp.pdf]

Supporting Information for

Neonatal diethylstilbestrol exposure disrupts uterine epithelial apical-basal polarity and partial EMT state

Rachel E. Bainbridge[1], Wendy N. Jefferson[1], Tianyuan Wang[2], Sara A. Grimm[2], and Carmen J. Williams[1,*]

*****Carmen Williams
Email: williamsc5@niehs.nih.gov

This PDF file includes:

Supporting text
Supplemental Figures S1 to S8
Legends for Datasets S1 to S15
SI References

Other supporting materials for this manuscript include the following:

Datasets S1 to S15

Supporting Information Text

Data Sharing

PND5 bulk RNA-seq data and PND5 histone H3K27ac ChIP-seq data are deposited in GSE104402 (1).

Data generated for this manuscript is made accessible through NLM BioProject.
Multiome: https://www.ncbi.nlm.nih.gov/bioproject/?term=prjna1259040 (2)
ERα ChIPseq: https://www.ncbi.nlm.nih.gov/bioproject/?term=prjna1259522 (3)
HiCseq: https://www.ncbi.nlm.nih.gov/bioproject/?term=prjna1259523 (4)

Supplemental Methods

Animal care, DES exposure, and tissue collection
Timed pregnant CD-1 mice were obtained from the in-house breeding colony at NIEHS, housed in a 12 h light:dark cycle, fed NIH-31 diet and water ad libitum. Following delivery, pups were randomly standardized to 10 female pups per dam. Pups were subcutaneously injected with DES (1 mg/kg/day; Sigma-Aldrich) or corn oil (vehicle control) for the first five days after birth. At PND5, mice were sacrificed 4 h after injection. Uteri for single nucleus multiomic sequencing were collected, pooled, fixed in Lomant's reagent [Dithiobis(succinimidyl propionate), DSP; ThermoFisher] and frozen to -70°C in a Mr. Frosty Freezing container (ThermoFisher), following the FixNCut protocol (5). Uteri collected for immunoblotting, ChIPseq or HiCseq were frozen immediately after collection on dry ice and stored at -70°C until use. Uteri collected for RNAscope, immunohistochemistry, or histology were fixed in 10% neutral buffered formalin. Uteri collected for epithelial isolation were collected into cold PBS (calcium and magnesium free, CMF) with protease inhibitors (Sigma).

Single nucleus multiomic sequencing
The nucleus extraction protocol was adapted from a previously published protocol for single-cell RNA-sequencing in DES-exposed adult mouse uterus (6). Fixed, frozen uterine tissue was crushed in a dry ice-cooled metal mortar and pestle, and 40-60 mg (uteri of approximately 10 mice per pooled sample) were used for nuclear isolation. Tissue homogenization was performed in Homogenization buffer (composition below) including NP40, digitonin, AlbuMAX bovine serum albumin (BSA), and Protector RNase inhibitor. The suspension was then incubated in OMNI Buffer RSB (composition below) with RNAse inhibitor on ice for 3 min. Nuclei were released by fragmentation in small and large cell dounce homogenizers. Nuclei were filtered through 100 μm and 30 μm CellTrics filters, centrifuged at 4000 rpm for 10 min at 4°C, then resuspended in 10X Nuclei buffer at a concentration of $1x10^6$ cells/mL for library preparation.

OMNI Buffer RSB

Reagent	Final Concentration
Tris-HCl (pH = 7.4)	10 mM
NaCl	10 mM
$MgCl_2$	3 mM
Tween-20	0.1%
	Store at 4°C

Homogenization buffer

Reagent	Final Concentration
NP40	0.1%
Digitonin	0.01%
BSA	0.9% BSA
RNase inhibitor	1 U/μL
OMNI RSB	Bring to desired volume

The 10X Genomics Chromium Next GEM Single Cell Multiome ATAC + Gene Expression Library Preparation Kit was used to generate a single nucleus barcoded library of mRNA and transposed DNA from individual nuclei. Single nuclei were counted on a Luna-Fx7 cell counter with AO/PI dyes and

examined for quality on a fluorescent microscope. About 16000 nuclei were loaded onto 10X Chip J followed by generating GEMs (Gel Bead-In Emulsions) on a 10X Chromium Controller. In each droplet of GEMs, mRNA was reverse transcribed and open chromatin was transposed with the same cell barcode. The reverse transcribed cDNA and transposed genomic DNA were amplified and separated to construct RNA-seq and ATAC-seq libraries following the instructions. The RNA-seq libraries were sequenced with parameters of 28 nt for read 1 and 90 nt for read 2. The ATAC-seq libraries were sequenced with parameters of 50 nt for read 1 and 49 nt for read 2. Sequencing was done on an Illumina NOVAseq 6000 with sequencing depth of $1x10^8$ read pairs/nucleus for the RNA-seq libraries and $1x10^8$ read pairs/nucleus for the ATAC-seq libraries.

Single nucleus multiomic analysis

Most analysis was performed with the Seurat (7) and Signac (8) packages. Cells were filtered for the following QC metrics: 500 < nCount_RNA < 10000, 500 < nCount_ATAC < 10000, 1 < TSS. enrichment < 20, 0.6 < nucleosome_signal < 0.25, percent.mt < 10, percent.rp < 15, pct_reads_in_peaks > 15. We performed normalization with SCTransform (9) and in some cases, integrated based on this transformation. Then, we performed dimensional reduction with principal component analysis (PCA) and uniform manifold approximation and projection (UMAP), followed by clustering based on shared nearest neighbor graph. Significantly differential genes were restricted to those with an adjusted p-value < 1×10^{-5}, average $\log_2$ fold change > |0.1|, and presence in >10% of cells in either exposure. RNA expression heatmaps were generated using Seurat or pheatmap (10). Ring plots were generated using scplotter (11). To assess cell-cell communication, LIANA was used (12). Pseudobulk ATAC signal tracks were generated after subsetting the CellRanger snATAC BAM file based on the CB (cell barcodes) tag. Heatmaps were generated using Seurat DoHeatmap (7) or DeepTools GenerateHeatmap (13). For the pseudobulk ATAC-seq heatmaps, the read counts in the DES sample were scaled to match the Control sample for reads-at-peaks (Control or DES counts/Control counts). Counts were considered the # of fragments overlapping the merged peak set, limited to reads that were on canonical chromosomes, properly paired, not PCR/optical duplicates, and were from cells that passed QC filters and were assigned to one of the seven cell types identified. The counts were normalized to the number of fragments aggregated over all cell types that were overlapping the merged peak set (N=109,028) to mitigate differences in signal:noise and total depth between samples. A scaling factor of 0.770 was applied to the DES sample to normalize it to the Control sample. In the bulk ERα ChIP-seq heatmaps, counts were normalized to 10 million mapped and deduplicated fragments per sample.

Artificial Intelligence (AI) Disclosure

The authors declare the use of generative artificial intelligence (GAI) in the research and writing process. According to the GAIDeT taxonomy (2025), the following tasks were delegated to GAI tools under full human supervision: Code generation, Code optimization. The GAI tool used was Claude 3. In addition, Claude 3 was used for code troubleshooting and some initial script writing. Responsibility for the final manuscript lies entirely with the authors.

EMT analysis

Partial EMT state was scored using a list of partial EMT marker genes (14), converted to mouse orthologs using the HUGO Gene Nomenclature Committee Comparison of Orthology Predictions search tool (https://www.genenames.org/tools/hcop/). Partial EMT score was calculated using Seurat AddModuleScore (7).

ERα ChIPseq

Uteri collected and fresh flash frozen from PND5 mice (140 mg of tissue per pooled sample group) were sent to Active Motif for ERα ChIPseq. ChIPseq reads (75 nt, single-end) were generated by Illumina sequencing (NextSeq 500). Raw single-end ChIPseq reads (75 bp) were filtered to retain reads with an average Phred quality score >20 and aligned to the mouse reference genome (mm10) using Bowtie v1.1.2 with unique mapping and allowing up to two mismatches per read (-m 1 -v 2) (15). PCR duplicates with identical alignment coordinates were removed using the MarkDuplicates function from Picard Tools. The retained reads were extended to 300 bp for downstream analyses. To normalize sequencing depth across ChIPseq datasets, reads were downsampled to the same number of uniquely mapped reads per sample. A single pooled input sample for diestrus and estrus was generated to verify antibody specificity but was not used for normalization. For visualization, bedGraph files were converted to bigWig format

using bedGraphToBigWig (16). The read coverage was displayed as custom tracks on the IGV Genome Browser, then manually overlayed to match Signac coverage plots in Adobe Illustrator.

Peak calling was performed using MACS2 with an adjusted p-value cutoff of <0.0001 (17). Differential regions between a pair of ChIPseq peaks were identified using MEDIPS (Model-based Exploration of DiPloid Sequencing data) with a window size of 250 bp (18). Differential analysis was performed using the medips.meth function with false discovery rate (FDR) adjustment (p.adj = "fdr") and edgeR as the statistical method (diff.method = "edgeR"). Differential regions were defined as genomic intervals exhibiting at least a two-fold difference in mapped read counts and an adjusted p-value ≤ 0.01.

HiCseq
Uterine tissues from PND5 control (16 uteri; 54 mg) and DES (5 uteri; 51 mg) were pooled and pulverized on dry ice. Instructions from the Arima Hi-C kit were followed. Sequencing was performed on an Illumina NOVAseq 6000. Raw paired-end Hi-C reads (151 bp) were aligned to the mouse reference genome (mm10) and processed using HiCUP v0.7.1 (19) for read truncation, filtering, and PCR duplicate removal. Only uniquely mapped di-tags that passed quality control and had an intra-chromosomal interaction distance greater than 10 kb were retained for downstream analyses. Chromatin loops were identified using the hiccups algorithm implemented in Juicer v1.8.9 with default parameters (20). The classification of common and differential loops was based on a loop's presence following DES exposure and in controls.

Immunohistochemistry

Target	Staining technique	Epitope retrieval	Non-specific site block	Primary antibody	Secondary antibody
Laminin	Avidin-biotin-peroxidase	Proteinase K 5 min at room temperature	10% normal donkey serum	Rabbit monoclonal laminin antibody (1.0 mg/ml) at a 1:7500 dilution or concentration matched normal rabbit IgG for 1 h at RT	Biotinylated donkey anti-rabbit IgG at a dilution of 1:500 for 30 min at RT
E-cadherin	Avidin-biotin-peroxidase	Heat induced in the Decloaker® pressure chamber for 15 min at 110°C in citrate buffer (pH 6.0)	10% normal horse serum	Mouse monoclonal E-cadherin antibody (0.25 mg/ml) or a concentration matched normal mouse IgG2a at a 1:1000 dilution for 1 h at RT	Biotinylated horse anti-mouse IgG at a dilution of 1:1000
ZO-1	Avidin-biotin-peroxidase	Heat induced in the Decloaker® pressure chamber for 15 min at 110°C in EDTA buffer solution (pH 8.5)	10% normal horse serum	Mouse monoclonal ZO-1 antibody (1.0 mg/ml) at a 1:200 dilution or concentration matched normal mouse IgG1 for 1 h at RT	Biotinylated horse anti-mouse IgG at a dilution of 1:1000

Immunohistochemical staining was performed using the techniques specified in the table above. Formalin-fixed, paraffin-embedded whole uterus tissue sections were deparaffinized in xylene and rehydrated through graded ethanol. Enzyme-induced epitope retrieval was performed. Endogenous peroxidase blocking was done by immersing the sections in 3% H_2O_2 for 15 min. Non-specific sites were blocked by incubating slides for 20 min in serum. Endogenous biotin and avidin binding sites were

blocked using an avidin-biotin blocking kit. Slides were incubated with primary antibody, then secondary antibody. Labeling incubation was done with the Vectastain RTU Kit Label for 30 min at RT. The antigen-antibody complex was visualized using 3-diaminobenzidine (DAB) chromogen for 6 min. Slides were counterstained with modified Harris hematoxylin, dehydrated through graded ethanol and coverslipped. Images were scanned using a Hamamatsu NanoZoomer S360 Digital Slide Scanner. Gamma correction was performed in NDP.view 2 (Hamamatsu) to level 1.8 unless otherwise stated in figure legend.

RNAscope

Formalin-fixed, paraffin-embedded whole uterine horns from control and DES-exposed mice were deparaffinized in xylene, rehydrated, blocked, and probed according to the RNAscope Multiplex Fluorescent Reagent Kit v2 instructions (Document number UM 323100, Advanced Cell Diagnostics, Inc). Opal 570 was used with the *Lgr5* probe on channel 1 (Advanced Cell Diagnostics, Inc), and Opal 690 with the *Esr1* probe on channel 2. Tissues were imaged with a Zeiss LSM 980 inverted confocal microscope with AiryScan.

Histology and F-actin staining

Whole uterine horns from control and DES-exposed mice were fixed in 10% neutral buffered formalin for approximately 48 h, moved to 70% ethanol, processed and embedded in paraffin blocks and sectioned at 5 μm thickness. For F-actin staining, following deparaffinization in xylene and ethanol, tissues were permeabilized with 0.1% Triton X-100 for 10 min. They were then incubated in 1:1000 phalloidin-rhodamine, washed, and counterstained with DAPI. Finally, tissues were imaged with a Zeiss LSM 980 inverted confocal microscope with AiryScan. Masson's Trichrome staining was performed according to standard procedures (21). Images were scanned using a Hamamatsu NanoZoomer S360 Digital Slide Scanner (Hamamatsu Corporation, Hamamatsu City, Japan).

Epithelial cell isolation

PND5 control and DES exposed uteri were collected into cold PBS-CMF + protease inhibitors (PBS-CMF+PI; Sigma; 1 tablet in 50 mL of PBS-CMF) (5 uteri were pooled per sample). Epithelial sheets were isolated using an adaptation of the protocol from (22). Uteri were incubated with 500 µL 1% trypsin (Gibco; 2.5% diluted to 1% with PBS-CMF+PI) for 30 minutes at 37°C in a Thermomixer at 900 rpm. Trypsin activity was stopped by adding 500 µL heat inactivated fetal bovine serum (FBS; Gibco) on ice for 5 min. Under a dissecting scope, epithelium was pulled out of the uterus by applying suction through glass Pasteur pipets pulled over a flame to approximately 100 µm diameter for controls and 150 µm for DES. Epithelial sheets were collected into a microcentrifuge tube and centrifuged at 10,000 rpm at 4°C for 5 min. The supernatant was removed and pellet was washed by adding cold PBS-CMF+PI and repeating the centrifuge step. Washing was performed three times to ensure trypsin and FBS were removed from samples. Epithelial cell pellets were frozen at -70°C until protein isolation.

Immunoblotting

PND5 whole frozen uteri (n=4 samples; 1 uterus per sample) or epithelial pellets (n=4 samples; pool of epithelial sheets from 5 mice per sample) were used to isolate cytoplasmic and nuclear fractions using the NE-PER kit (ThermoFisher). Protein concentration was quantified using a Qubit protein assay (ThermoFisher). Proteins were loaded on a stain free 4-20% TGX 10-well gel (Bio-Rad) using 10 µg per lane for whole uterus and epithelial enriched cytoplasmic fraction, 5 µg for whole uterus, and 2.5 µg for epithelial nuclear fraction. Proteins were transferred to low fluorescence PVDF membrane (Bio-Rad) using a Trans-Blot Turbo transfer system (Bio-Rad) on the mixed molecular weight setting. Blots were incubated with 5% Blotto, non-fat milk (Santa Cruz) in tris buffered saline (TBS; Bio-Rad) with 1% Tween-20 (Sigma; TBS-T). Blots were then incubated with anti-active beta catenin (Cell Signaling) at a dilution of 1:1000 in 5% milk in TBS-T overnight at 4°C in roller bottles. Blots were washed three times for 15 min with TBS-T on a shaker. Blots were then incubated with peroxidase anti-rabbit IgG made in a donkey (Jackson ImmunoResearch) diluted 1:25,000 in 1% milk in TBS-T for 1 h at room temperature on a rocker. Blots were washed as above and signal detected using SuperSignal West Femto (Fisher Scientific). Bands were detected using a Chemi-Doc imaging system (Bio-Rad). Blots were stripped using Restore (Fisher Scientific) for 30 min at 37°C. Immunoblotting for GAPDH was performed as described above using anti-GAPDH (Santa Cruz) at a dilution of 1:1000. Immunoblotting for actin was performed as

describe above except a single step was used for primary and secondary detection using peroxidase linked anti-actin (Sigma) at a dilution of 1:10,000 in 1% milk in TBS-T for 1 h at room temperature.

Key Reagents Table

REAGENT or RESOURCE	SOURCE	IDENTIFIER
**Antibodies**		
Laminin Polyclonal Antibody	ThermoFisher Scientific	Cat# PA1-16730, RRID:AB_2133633
E-cadherin Monoclonal Antibody	BD Biosciences	Cat# 610182, RRID:AB_397581
Active Beta Catenin antibody	Cell Signaling	Cat# 8814S, RRID:AB_11127203
GAPDH antibody	Sant Cruz	Sc-25778, RRID:AB_10167668
Peroxidase anti-actin antibody	Sigma	A3854, RRID:AB_262011
Peroxidase anti-rabbit made in a donkey	Jackson ImmunoResearch	711-035-152, RRID:AB_10015282
ZO-1 Recombinant Mouse Monoclonal Antibody (ZO1-1A12)	ThermoFisher Scientific	Cat# 740002M, RRID:AB_3074173
ERα antibody	Santa Cruz	Cat# sc-543
Normal rabbit IgG	Abcam	Cat# ab171870, RRID:AB_2687657
Normal mouse IgG2a	BD Pharmingen	Cat# 553501
Normal mouse IgG1	BD Pharmingen	Cat# 555746
Biotinylated donkey anti-rabbit IgG antibody (1:500 dilution)	Jackson ImmunoResearch	Cat#711-066-152, RRID:AB_2340594
Biotinylated horse anti-mouse IgG antibody (1:1000 dilution)	Vector Laboratories	Cat#BA-2000-1.5, RRID:AB_2313581
Rabbit on Rodent HRP Polymer	Biocare	Cat# RMR622, lot 100923A
**Chemicals, peptides, and recombinant proteins**		
RNAscope™ Multiplex Fluorescent Detection Kit v2	ACD	Cat# 323110
RNAscope pretreatment reagents (Target retrieval, Pretreatment 2)	ACD	Cat# 322000
RNAscope pretreatment reagents (Hydrogen peroxide, Protease Plus, Protease III, and Protease IV)	ACD	Cat# 322381
RNAscope Wash buffer reagents	ACD	Cat# 310091
RNAscope® Probe - Mm-Lgr5	ACD	Cat# 312171
RNAscope® Probe - Mm-Esr1-C2	ACD	Cat# 478201-C2
Opal 570 Reagent Pack	Akoya Biosciences	FP1488001KT
Opal 690 Reagent Pack	Akoya Biosciences	FP1497001KT
Phalloidin-rhodamine	ThermoFisher Scientific	Cat# R415
Bouin's Solution	Lab Chem Inc.	Cat# LC11790-1
Hematoxylin crystals	Chroma-Gesellschaft	Cat# 5B 535
Ferric Chloride, 29% aqueous	ThermoFisher Scientific	Cat# 188-100
Hydrochloric acid, concentrated	ThermoFisher Scientific	Cat# A508-500
Biebrich scarlet	Rowley Biochemical	CAS: 4196-99-0
Acid fuchsin	Chroma-Gesellschaft	Cat# 1B 525
Phosphomolybdic acid	ThermoFisher Scientific	Cat# A237-100

Phosphotungstic acid	ThermoFisher Scientific	Cat# A248-100
Aniline blue	ThermoFisher Scientific	Cat# A-967
Glacial acetic acid	ThermoFisher Scientific	Cat# A35-500
Light green, SF yellowish	Rowley Biochemical	CAS: 5141-20-8
Diethylstilbestrol (DES)	Sigma-Aldrich	CAS: 56-53-1
Corn oil (vehicle control)	Spectrum Chemical	CAS: 8001-30-7
Dithiobis(succinimidyl propionate) (DSP), Lomant's reagent	ThermoFisher	Cat# 22586
Protector RNase inhibitor	Sigma-Aldrich	SKU 3335402001
Proteinase K	Leica Biosystems	Cat# RE7160-K
3-diaminobenzidine (DAB) chromogen	Dako	N/A
Normal donkey serum	Jackson ImmunoResearch	Cat# 017-000-121
Normal horse serum	Jackson ImmunoResearch	Cat# 008-000-121
Antigen Decloaker, 10X, Citrate buffer (pH 6.0)	Biocare Medical	Cat#CB910M
EDTA buffer solution (pH 8.5)	Biocare Medical	Cat# CB917
2.5% Trypsin	Gibco	Cat# 15090-046
Heat inactivated fetal bovine serum	Gibco	Cat# 6505
Tween-20	Sigma	Cat# P1379
TBS	Bio-Rad	Cat# 1706435
Blotto – non-fat dry milk	Santa Cruz	Cat# sc-2325
Protease inhibitor	Sigma	Cat# 46548400
**Kits**		
10X Genomics Chromium Next GEM Single Cell Multiome ATAC + Gene Expression Library Preparation Kit	10X Genomics	Cat# 1000285
Vectastain RTU Kit Label	Vector Laboratories	Cat# PK-7200
Avidin-biotin blocking kit	Vector Laboratories	Cat# SP-2001
Arima Hi-C kit	Arima	Cat# A510008
NE-PER kit	ThermoFisher	Cat# 78833
Qubit protein quantification kit	Fisher Scientific	Cat# A50668
SuperSignal West Femto kit	Fisher Scientific	Cat# 34095
**Experimental models: Organisms/strains**		
CD-1 mice (timed pregnant)	NIEHS in-house breeding colony	N/A
**Oligonucleotides**		
See RNAscope probes above		
**Sequence-based reagents**		
See RNAscope probes above		
**Software and algorithms**		
R	R Project	v4.3.2; https://www.r-project.org/
Python	Python Software Foundation	v3.9.6 & v3.11.6; https://www.python.org/
NumPy	Harris et al (23)	v1.26.4

pandas	The pandas development team (24)	v2.1.1
SciPy	Virtanen et al (24)	v1.15.1
anndata	Virshup et al (25)	v0.10.9
matplotlib	Hunter (26)	v3.9.4
scanpy	Wolf et al (27)	v1.10.3
seaborn	Waskom (28)	v0.13.2
NDP.view2Plus	Hamamatsu	https://www.hamamatsu.com/
BedGraphToBigWig	Kent et al (16)	v2.10
samtools	Li et al (29)	v1.18
bedtools	Quinlan & Hall (30)	v2.31.1
deepTools	Ramírez et al (13)	v3.5.1
pheatmap	Kolde (10)	v1.0.12
UpSetR	Conway et al (31)	v1.4.0
MEDIPS	Lienhard et al (18)	v1.54.0
Rsamtools	Morgan et al (32)	v2.18.0
scplotter	Wang (11)	v0.1.1
readxl	Wickham & Bryan (33)	v1.4.3
liana	Dimitrov et al (12)	v0.1.13
BSgenome.Mmusculus.UCSC.mm10	Team TBD (34)	v1.4.3
BSgenome	Pagès (35)	v1.70.2
BiocIO	Morgan et al (36)	v1.12.0
Biostrings	Pagès et al (37)	v2.70.3
XVector	Pagès & Aboyoun (38)	v0.42.0
EnsDb.Mmusculus.v79	Rainer (39)	v2.99.0
ensembldb	Rainer et al (40)	v2.26.0
AnnotationFilter	Morgan & Rainer (41)	v1.26.0
GenomicFeatures	Lawrence et al (42)	v1.54.4
SparseM	Koenker & Ng (43)	v1.84-2
AnnotationDbi	Pagès et al (44)	v1.64.1
graph	Gentleman et al (45)	v1.80.0
limma	Ritchie et al (46)	v3.58.1
stringr	Wickham (47)	v1.5.1
dplyr	Wickham et al (48)	v1.1.4
readr	Wickham et al (49)	v2.1.5
tibble	Müller & Wickham (50)	v3.2.1
tidyverse	Wickham et al (51)	v2.0.0
scMerge	Lin et al (52)	v1.18.0
scran	Lun et al (53)	v1.30.2
scuttle	McCarthy et al (54)	v1.12.0
MAST	Finak et al (55)	v1.28.0
SingleCellExperiment	Amezquita et al (56)	v1.24.0
SummarizedExperiment	Morgan et al (57)	v1.32.0
Biobase	Huber et al (58)	v2.62.0
GenomicRanges	Lawrence et al (42)	v1.54.1

GenomeInfoDb	Arora et al (59)	v1.38.8
IRanges	Lawrence et al (42)	v2.36.0
S4Vectors	Pagès et al (60)	v0.40.2
BiocGenerics	Huber et al (58)	v0.48.1
MatrixGenerics	Ahlmann-Eltze et al (61)	v1.14.0
matrixStats	Bengtsson (62)	v1.5.0
tidyr	Wickham et al (51)	v1.3.1
patchwork	Pedersen (63)	v1.2.0
ggrepel	Slowikowski (64)	v0.9.6
ggplot2	Wickham (65)	v3.5.2
irlba	Baglama & Reichel (66)	v2.3.5.1
Signac	Stuart et al (8)	v1.14.0
SeuratDisk		v0.0.0.9021
Seurat	Hao et al (7)	v5.3.0
SeuratObject	Satija et al (67)	v5.1.0
sp	Pebesma & Bivand (68) Bivand et al (69)	v2.2-0
Bowtie	Langmead et al (15)	v1.1.2
MACS	Zhang et al (17)	
HOMER	Heinz et al (70)	v4.5
IGV genome browser	Robinson et al (71)	v2.18.4
HiCUP	Wingett (19)	v0.7.1
Juicer	Durand et al (20)	v1.8.9
SCTransform	Choudhary & Satija (9)	
Claude	Anthropic	https://claude.ai
**Other**		
NIH-31 diet	N/A	N/A
100 µm CellTrics filters	Fisher Scientific	Cat# 04-0042-2318
30 µm CellTrics filters	Fisher Scientific	Cat# 04-0042-2316
Mr. Frosty Freezing container	ThermoFisher	N/A
10X Chip J	10X Genomics	N/A
Small cell dounce homogenizer	Kontes Glass Co.	N/A
Large cell dounce homogenizer	Kontes Glass Co.	N/A
10X Chromium Controller	10X Genomics	N/A
Illumina NOVAseq 6000	Illumina	N/A
Illumina NextSeq 500	Illumina	N/A
Hamamatsu NanoZoomer S360 Digital Slide Scanner	Hamamatsu Corporation	N/A
Zeiss LSM 980 inverted confocal microscope with AiryScan	Zeiss	N/A
Decloaker® pressure chamber	Biocare Medical	N/A
Luna-Fx7 cell counter with AO/PI dyes	Logos Biosystems	N/A
Stain free 4-20% TGX gels	Bio-Rad	Cat# 4561094
PVDF – low fluorescence	Bio-Rad	Cat# 1620260

Supplemental Figures

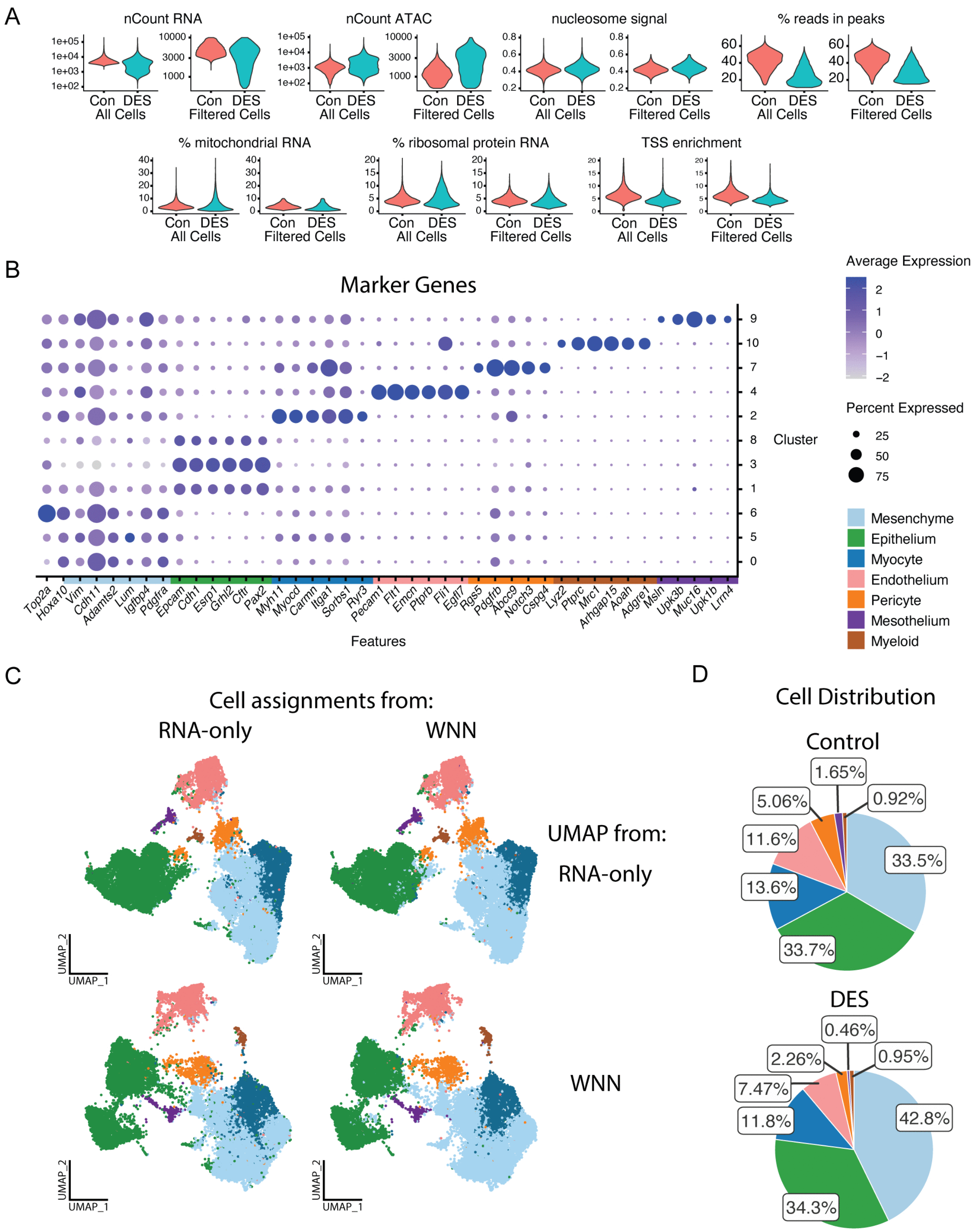

Figure S1. Manual annotation of integrated cells from control and DES-exposed uteri. A) Violin plots of QC metrics for control and DES exposed uterine cells before and after filtering. B) Dot plot of markers for proliferative cells (*Top2a*), mesenchyme (*Hoxa10, Vim, Cdh11, Adamts2, Lum, Itgbp4, Pdgfra*), epithelium (*Epcam*, *Cdh1, Esrp1, Grhl2, Cftr, Pax2*), myocyte (*Myh11, Myocd, Carmn, Itga1, Sorbs1, Ryr3*), endothelium (*Pecam1, Flt1, Emcn, Ptprb, Fli1, Egfl7*), pericyte (*Rgs5, Pdgfrb, Abcc9, Notch3, Cspg4*), mesothelium (*Msln, Upk3b, Muc16, Upk1b, Lrrn4),* and myeloid (*Lyz2, Ptprc, Mrc1,*

Arhgap15, Aoah, Adgre1). C) Comparison of cell assignments from RNA-only or weighted nearest neighbor (WNN) analysis, where UMAP coordinates are from RNA-only in top row or WNN in bottom row, and cell assignments are from RNA-only in the left column and WNN in the right column. D) Relative composition of control (top) and DES-exposed (bottom) uterine cells as annotated in Figure 1.

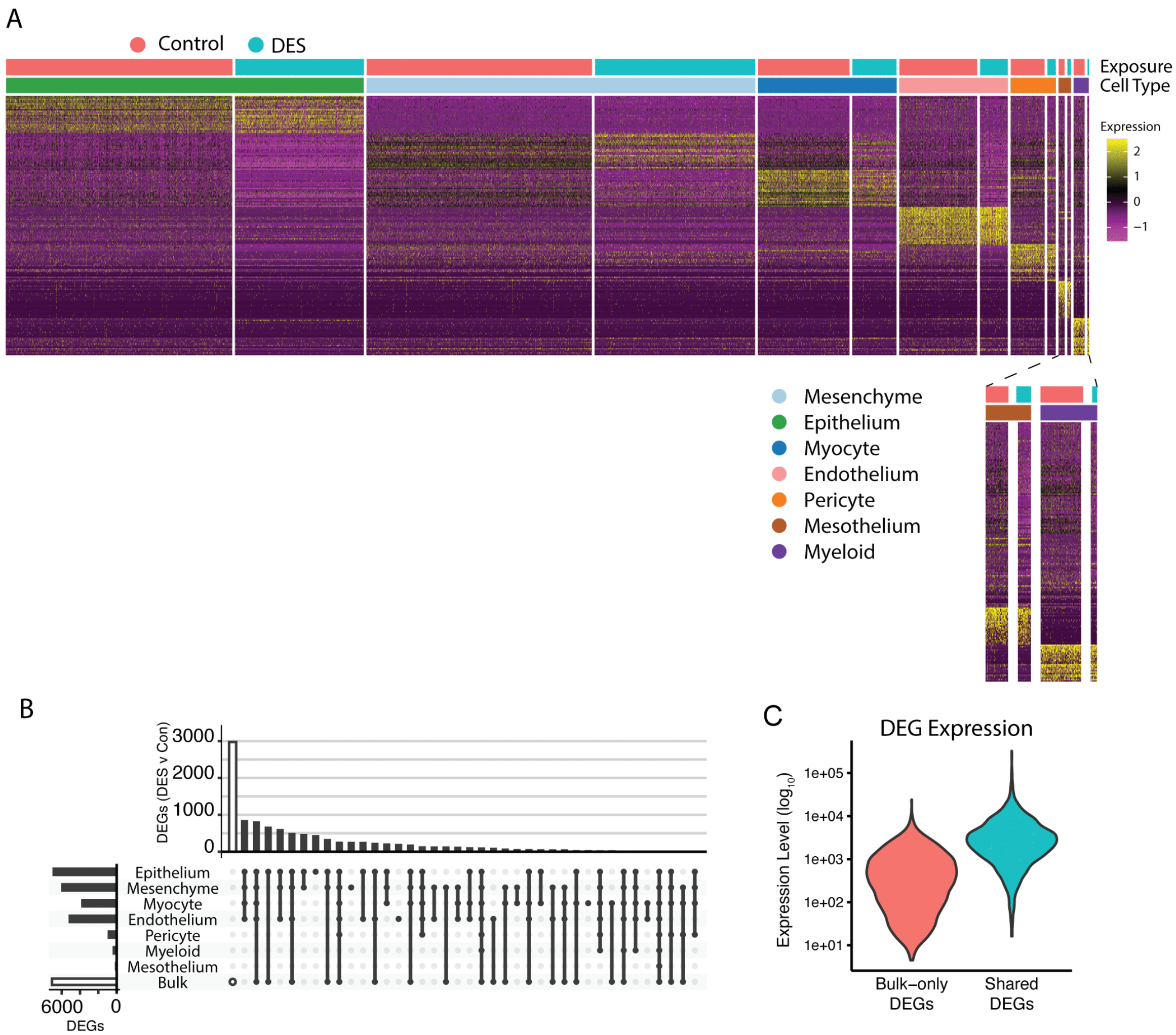

Figure S2. Gene expression differences between control and DES-exposed uterine cell types and comparison to bulk RNAseq. A) Heatmap of RNA expression across all cells of top marker genes for each cell type per exposure. Expanded view of mesothelial and myeloid cells shown below heatmap. B) UpSet plot of differentially expressed genes between DES-exposed and control cells in each cell type or previously published bulk sequencing from dataset GSE104402 (1, 72). C) Expression level of differentially expressed genes appearing only in bulk RNAseq vs those appearing both in bulk and snRNAseq (shared).

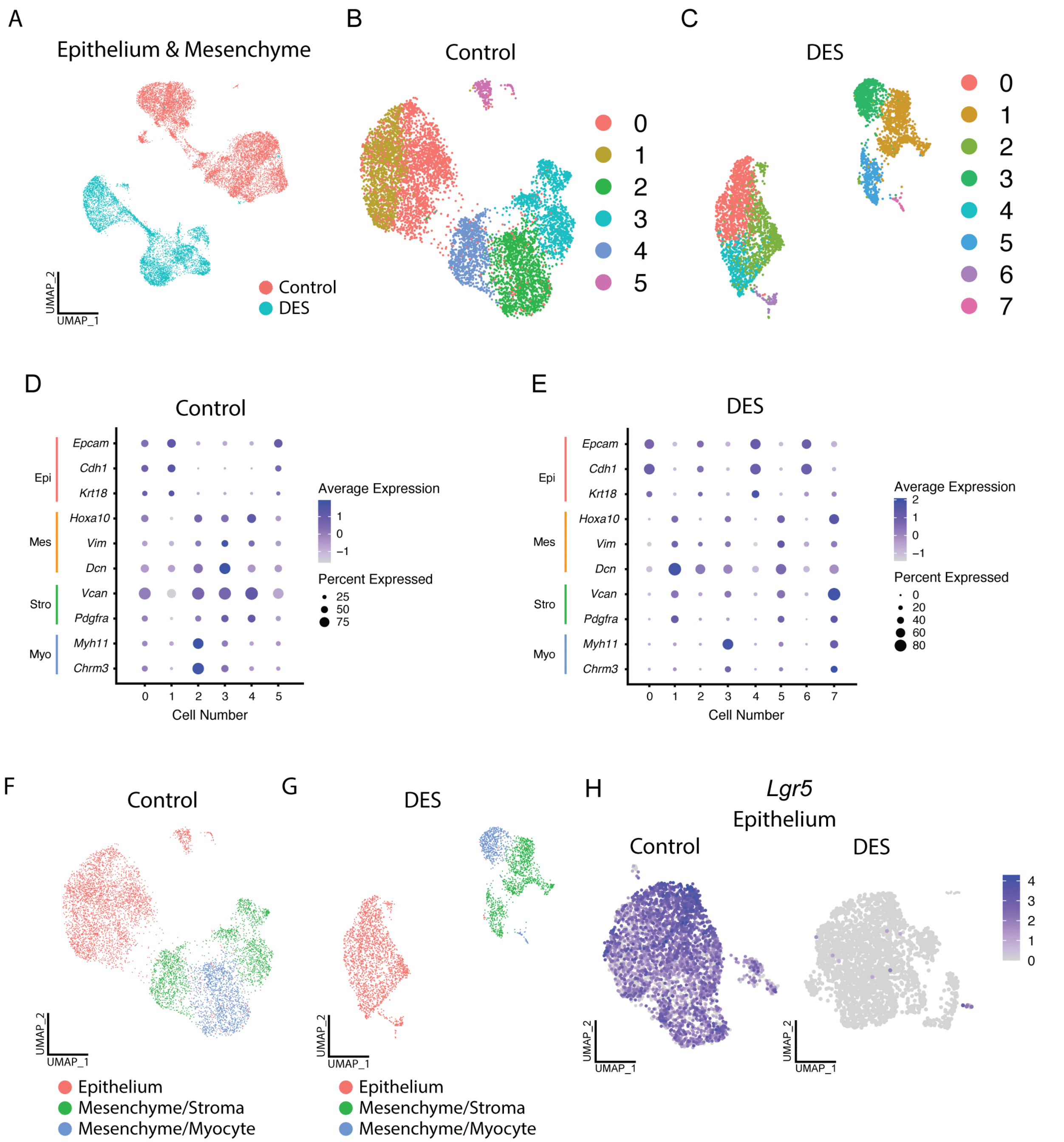

Figure S3. Manual annotation of nonintegrated cells from control and DES-exposed uterine epithelium and mesenchyme. A) UMAP of integrated epithelium, mesenchyme, and myocytes from both control and DES-exposed uterus, labeled by exposure. B) UMAP of integrated control uterine epithelium, mesenchyme, and myocytes labeled by cluster. C) UMAP of integrated DES-exposed uterine epithelium, mesenchyme, and myocytes labeled by cluster. D) Dot plot of expression of markers for myocyte (*Myh11, Chrm3*), stroma (*Vcan, Pdgfra*), epithelium (*Epcam*, *Cdh1*, *Krt18*), and mesenchyme (*Hoxa10, Vim, Dcn*) in control uterine epithelium and mesenchyme. E) Dot plot of expression of same markers in DES-exposed epithelium and mesenchyme. F-G) Annotated UMAPs of integrated control (F) and DES-exposed (G) uterine epithelium, mesenchyme with additional stromal markers, and mesenchyme with additional myocyte markers. H) Feature plots of *Lgr5* expression in control and DES epithelium (see Figure 1D).

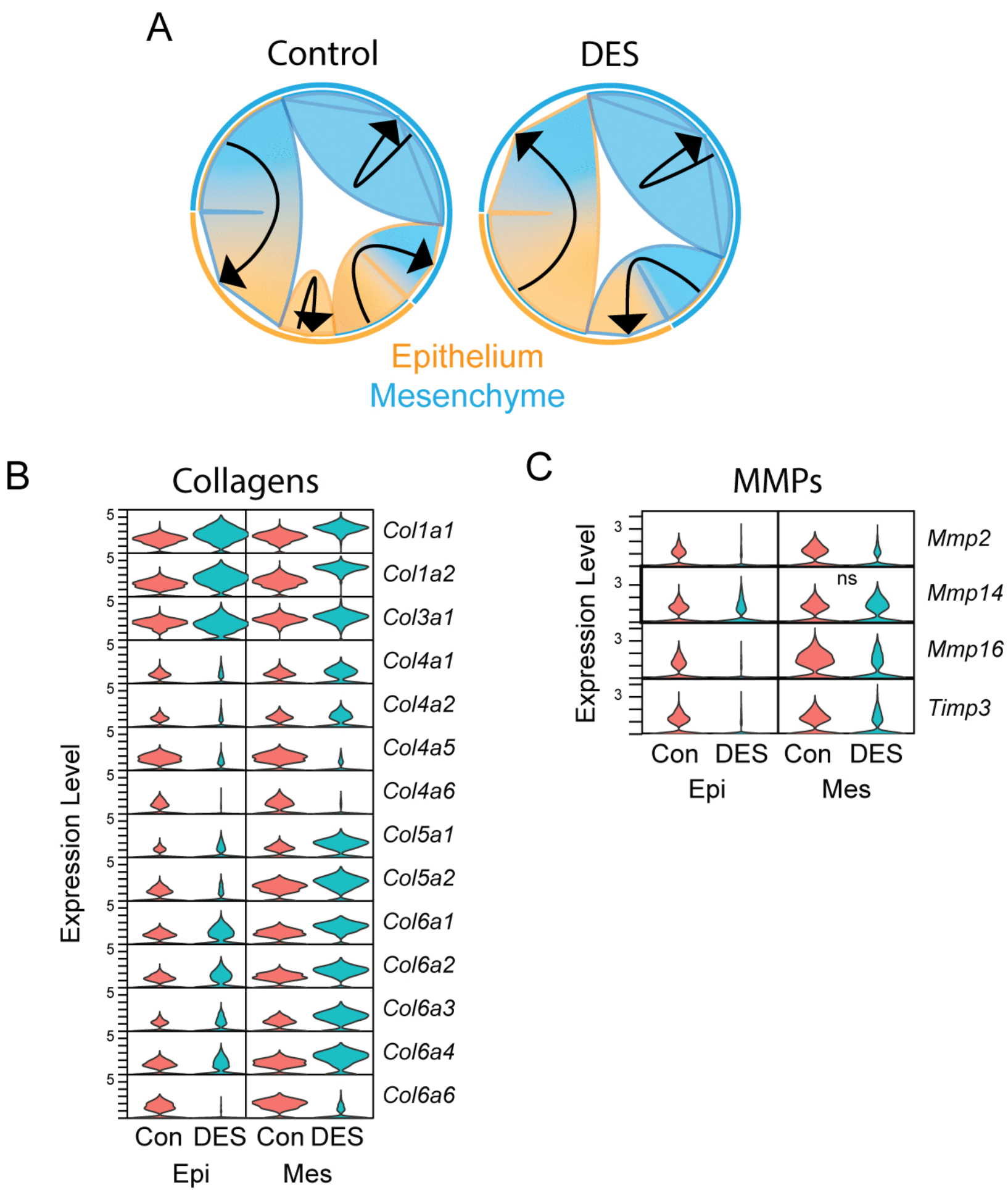

Figure S4. Altered uterine epithelial-mesenchymal communication and gene expression following DES exposure. A) Chord map depicting relative abundance of possible ligand-receptor pairs between mesenchyme-mesenchyme (blue), mesenchyme-epithelium (blue to orange), epithelium-mesenchyme (orange to blue), and epithelium-epithelium (orange). Arrow directions are from source to target. B) Violin plots of RNA expression of collagen genes expressed in control or DES-exposed epithelium or mesenchyme. C) Violin plots of RNA expression of matrix metalloprotease genes in control or DES-exposed epithelium or mesenchyme. Gene expression in all violin plots is significantly different unless marked as “ns”.

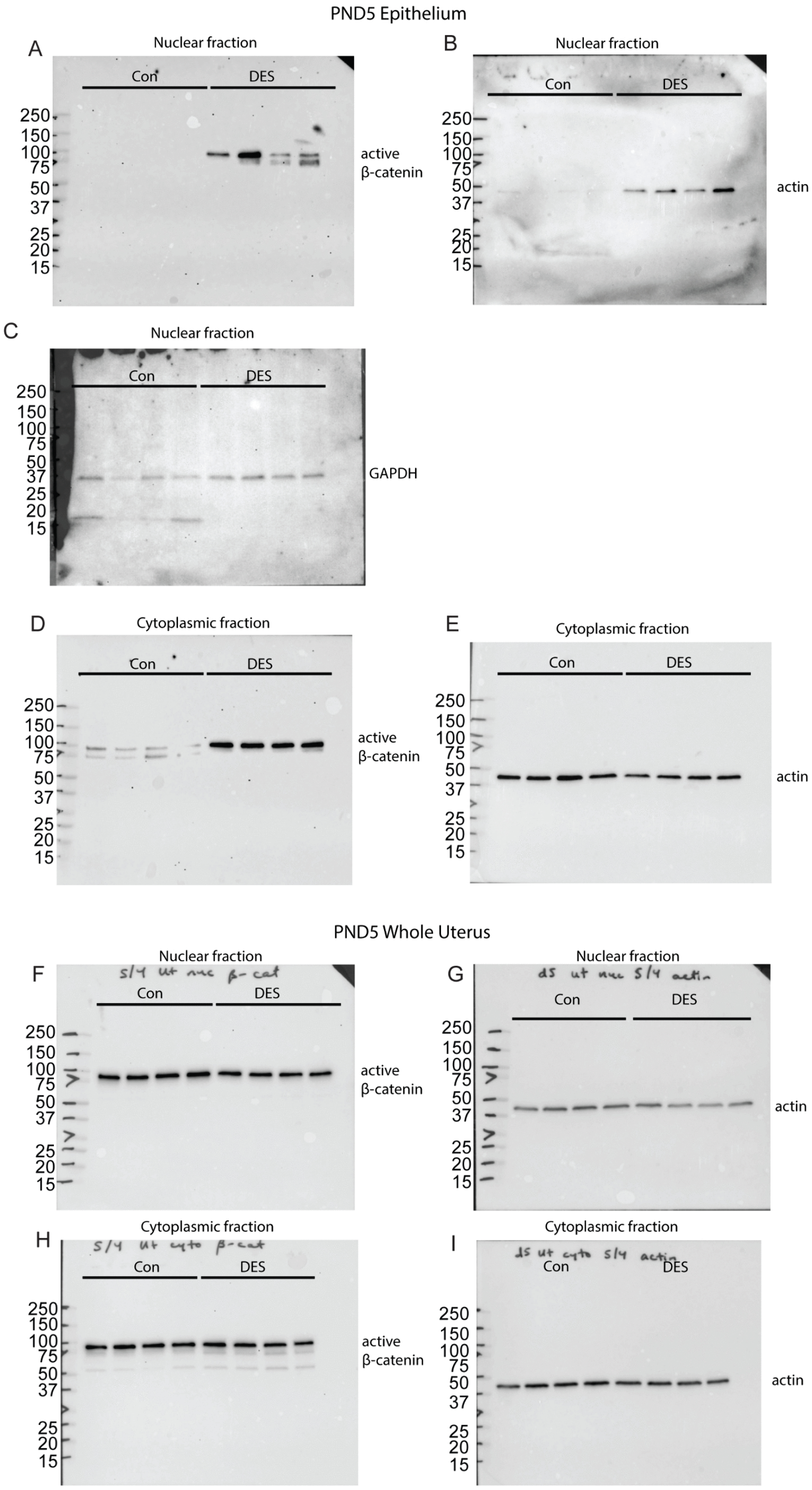

Figure S5. Uncropped immunoblots of active β-catenin, actin, and GAPDH in PND5 uterine cells with or without DES exposure. A-E) Immunoblots of the nuclear (A-C) or cytoplasmic (D-E) fractions of uterine epithelial protein immunoblotted for active β-catenin (A,D), actin (B,E) or GAPDH (C). F-I) Immunoblots of the nuclear (F,G) or cytoplasmic (H,I) fractions of total uterine protein stained for active β-catenin (F,H) or actin (G,I). Epithelium was pooled from 5 uteri per sample from control and DES-exposed mice (N = 4 samples per group). C) Individual uteri from control and DES-exposed mice (N=4 uteri per group).

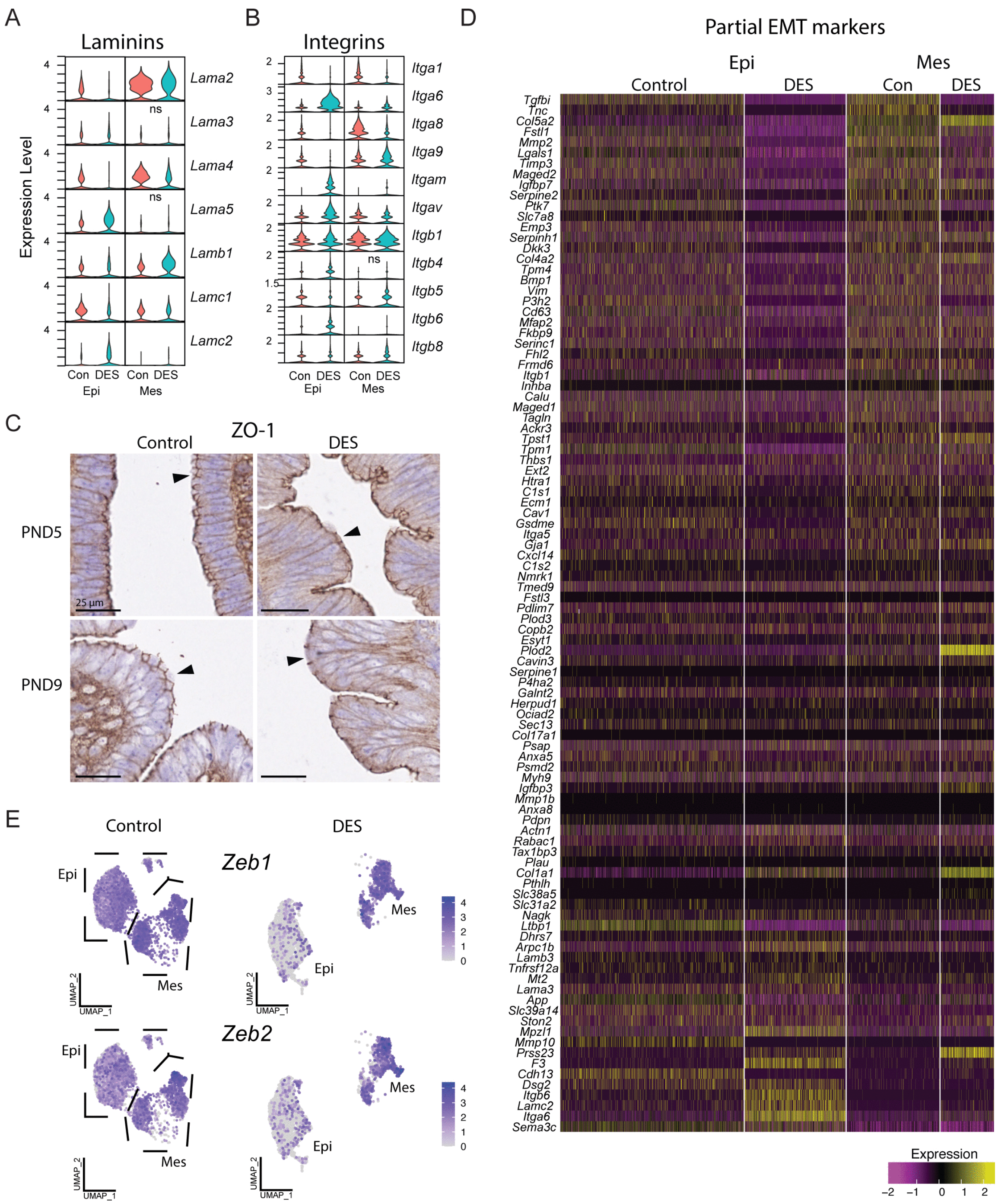

Figure S6. Reduction in partial EMT gene expression in DES-exposed epithelium. A-B) Violin plots of RNA expression of laminin (A) or integrin (B) genes in control and DES-exposed epithelium and mesenchyme. Gene expression in all violin plots is significantly different unless marked as "ns". C) Representative images of immunohistochemical staining of tight junction protein ZO-1 in control and DES-exposed uterus at PND5 (Control N = 8 from 5 mice, DES N = 9 from 5 mice). D) Expanded view of heatmap of pro-partial EMT markers (10) in control or DES-exposed epithelium and mesenchyme showing genes included. E) Feature plots of EMT transcription factors *Zeb1* and *Zeb2* in control and DES exposed epithelium and mesenchyme (see Figure S2).

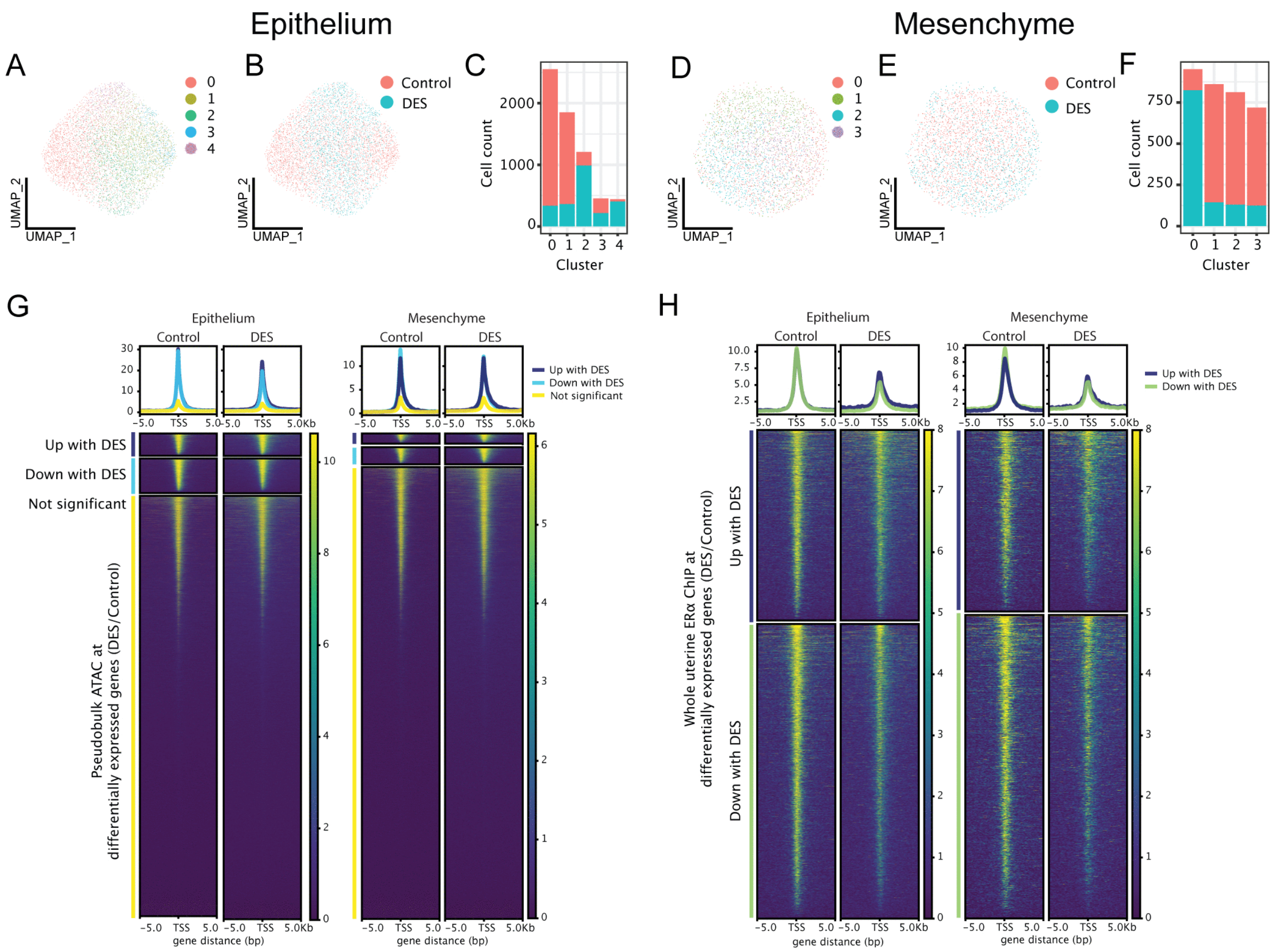

Figure S7. Alterations in chromatin accessibility following DES exposure. A) UMAP of epithelial cells from control or DES-exposed uteri with clustering based on snATACseq data. B) Epithelial ATAC UMAP labeled by exposure. C) Stacked plot representing the number of cells from control or DES exposed epithelium in each cluster. D) UMAP of mesenchymal cells from control or DES-exposed uteri with clustering based on snATACseq data. E) Mesenchymal ATAC UMAP labeled by exposure. F) Stacked plot representing the number of cells from control or DES exposed mesenchyme in each cluster. G) Metaplots and heatmaps of pseudobulk, normalized ATAC signal at TSS of all GENCODE genes from control or DES-exposed epithelium and mesenchyme. H) Metaplots and heatmaps of whole uterine ERα ChIP reads at TSS from 2881 upregulated and 4326 downregulated genes altered in epithelium, and in 1321 upregulated and 2166 downregulated genes altered in mesenchyme, as indicated.

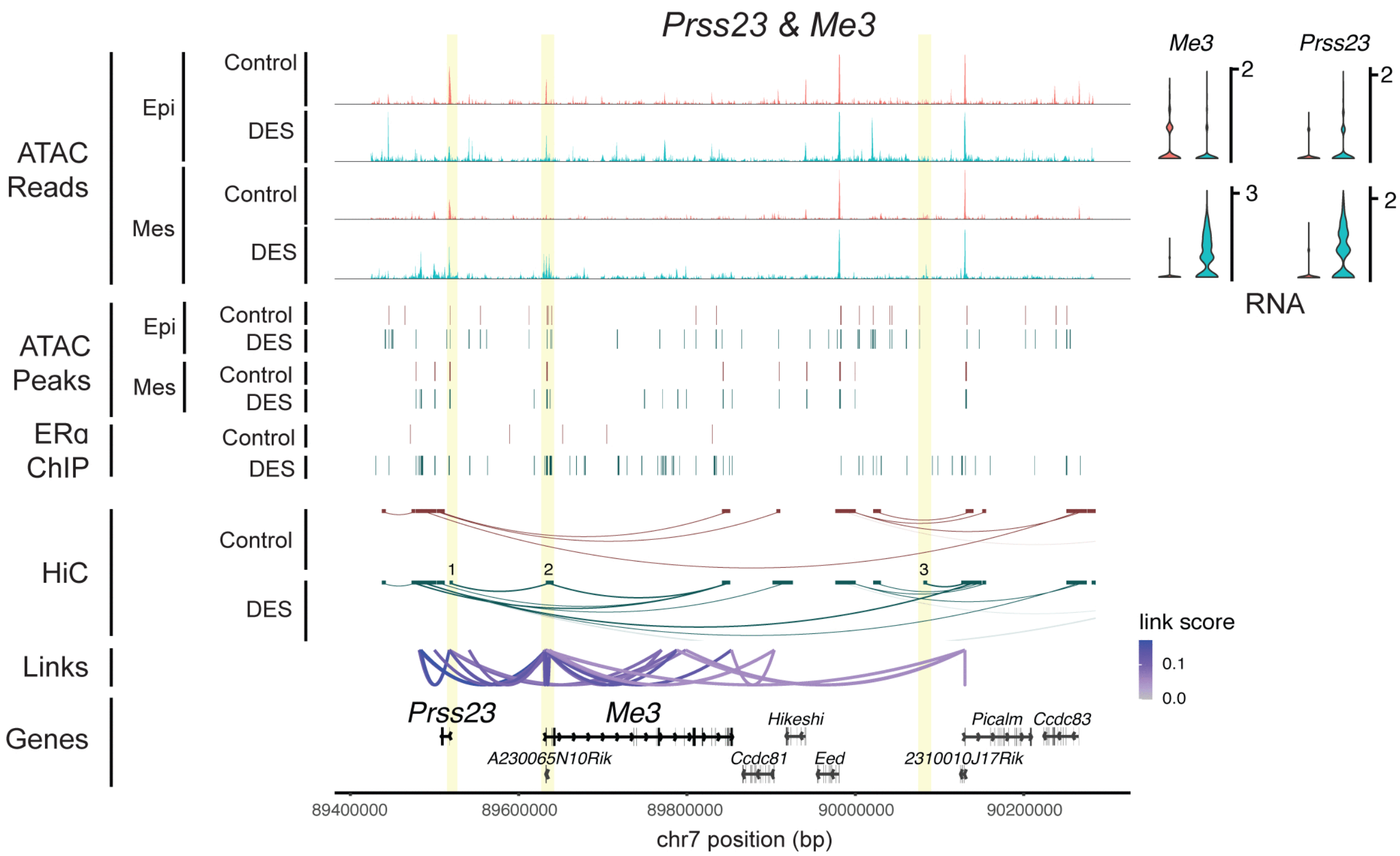

Figure S8. Alterations in chromatin accessibility, looping, and ERα binding co-occur at common sites in the DES-exposed uterine epithelium. Gene tracks at area surrounding *Prss23* and *Me3*, including normalized psuedobulk ATAC signal and peaks in control or DES-exposed uterine epithelial or mesenchymal cells, ERα ChIP peaks from control or DES-exposed whole uterus, HiCseq loops from control or DES-exposed whole uterus, and linkage of RNA expression to open chromatin based on cell-specific ATAC signal. Yellow bars denote areas of interest mentioned in main text. 1, DES-specific loop at *Prss23* promoter; 2, DES-specific loop at *Me3* promoter; 3, DES-specific loop upstream of *Picalm* with ERα binding site.

List of Datasets

Dataset S1 (separate file). Differentially expressed genes per cluster (whole uterus)

Dataset S2 (separate file). Differentially expressed genes per cell type and exposure (whole uterus)

Dataset S3 (separate file). Differentially expressed genes in integrated epithelial, mesenchymal, and stromal cells (DES/Control)

Dataset S4 (separate file). Differentially expressed genes per cluster (Control; epithelial and mesenchymal subset)

Dataset S5 (separate file). Differentially expressed genes per cluster (DES; epithelial and mesenchymal subset)

Dataset S6 (separate file). Epithelial differentially expressed genes (DES/Control)

Dataset S7 (separate file). Mesenchymal differentially expressed genes (DES/Control)

Dataset S8 (separate file). Liana top 50 ligand-receptor pairs as shown in Figure 3B

Dataset S9 (separate file). Liana all ligand-receptor pairs (Control epithelium & mesenchyme)

Dataset S10 (separate file). Liana all ligand-receptor pairs (DES epithelium & mesenchyme)

Dataset S11 (separate file). Differential ATAC peaks (DES/control epithelium)

Dataset S12 (separate file). Differential ATAC peaks (DES/control mesenchyme)

Dataset S13 (separate file). ERα ChIPseq peaks (DES whole uterus)

Dataset S14 (separate file). ERα ChIPseq peaks (Control whole uterus)

Dataset S15 (separate file). Differential HiC (DES/Control whole uterus)

SI References

1. W. N. Jefferson, Data from "ERα Dependent Alterations in the Epigenetic Landscape of the Mouse Uterus Following Neonatal Estrogen Exposure." NCBI GEO. https://www.ncbi.nlm.nih.gov/geo/query/acc.cgi?acc=GSE104402. Deposited 28 Sep 2017.
2. R. E. Bainbridge, Data from "Single nucleus multiomic sequencing of the neonatal mouse (PND5) uterus after exposure to diethylstilbestrol." NCBI BioProject. https://www.ncbi.nlm.nih.gov/bioproject/?term=PRJNA1259040. Deposited 5 May 2025.
3. R. E. Bainbridge, Data from "ERa ChIPseq of the neonatal mouse uterus after exposure to diethylstilbestrol." NCBI BioProject. https://www.ncbi.nlm.nih.gov/bioproject/?term=PRJNA1259522. Deposited 6 May 2025.
4. R. E. Bainbridge, Data from "HiCseq of the neonatal mouse uterus after exposure to diethylstilbestrol." NCBI BioProject. https://www.ncbi.nlm.nih.gov/bioproject/?term=PRJNA1259523. Deposited 6 May 2025.
5. L. Jimenez-Gracia *et al.*, FixNCut: single-cell genomics through reversible tissue fixation and dissociation. *Genome Biol* **25**, 81 (2024).
6. E. Padilla-Banks *et al.*, Developmental estrogen exposure in mice disrupts uterine epithelial cell differentiation and causes adenocarcinoma via Wnt/beta-catenin and PI3K/AKT signaling. *PLoS Biol* **21**, e3002334 (2023).
7. Y. Hao *et al.*, Dictionary learning for integrative, multimodal and scalable single-cell analysis. *Nat Biotechnol* **42**, 293–304 (2024).
8. T. Stuart, A. Srivastava, S. Madad, C. A. Lareau, R. Satija, Single-cell chromatin state analysis with Signac. *Nat Methods* **18**, 1333–1341 (2021).
9. S. Choudhary, R. Satija, Comparison and evaluation of statistical error models for scRNA-seq. *Genome Biol* **23**, 27 (2022).
10. R. Kolde (2025) pheatmap: Pretty Heatmaps.
11. P. Wang (scplotter: Publication Quality Plots for Single Cell Data Analysis. (GitHub).
12. D. Dimitrov *et al.*, Comparison of methods and resources for cell-cell communication inference from single-cell RNA-Seq data. *Nat Commun* **13**, 3224 (2022).
13. F. Ramirez *et al.*, deepTools2: a next generation web server for deep-sequencing data analysis. *Nucleic Acids Res* **44**, W160–165 (2016).
14. S. V. Puram *et al.*, Single-Cell Transcriptomic Analysis of Primary and Metastatic Tumor Ecosystems in Head and Neck Cancer. *Cell* **171**, 1611–1624 e1624 (2017).
15. B. Langmead, C. Trapnell, M. Pop, S. L. Salzberg, Ultrafast and memory-efficient alignment of short DNA sequences to the human genome. *Genome Biol* **10**, R25 (2009).
16. W. J. Kent, A. S. Zweig, G. Barber, A. S. Hinrichs, D. Karolchik, BigWig and BigBed: enabling browsing of large distributed datasets. *Bioinformatics* **26**, 2204–2207 (2010).
17. Y. Zhang *et al.*, Model-based analysis of ChIP-Seq (MACS). *Genome Biol* **9**, R137 (2008).
18. M. Lienhard, C. Grimm, M. Morkel, R. Herwig, L. Chavez, MEDIPS: genome-wide differential coverage analysis of sequencing data derived from DNA enrichment experiments. *Bioinformatics* **30**, 284–286 (2014).
19. S. Wingett *et al.*, HiCUP: pipeline for mapping and processing Hi-C data. *F1000Res* **4**, 1310 (2015).
20. N. C. Durand *et al.*, Juicer Provides a One-Click System for Analyzing Loop-Resolution Hi-C Experiments. *Cell Syst* **3**, 95–98 (2016).
21. L. G. Luna, *Manual of histologic staining methods of the Armed Forces Institute of Pathology* (McGraw-Hill, New York, ed. 3rd, 1968).
22. T. E. Spencer, M. T. Lowke, K. M. Davenport, P. Dhakal, A. M. Kelleher, Single-cell insights into epithelial morphogenesis in the neonatal mouse uterus. *Proc Natl Acad Sci U S A* **120**, e2316410120 (2023).
23. C. R. Harris *et al.*, Array programming with NumPy. *Nature* **585**, 357–362 (2020).
24. P. Virtanen *et al.*, SciPy 1.0: fundamental algorithms for scientific computing in Python. *Nat Methods* **17**, 261–272 (2020).
25. I. Virshup, S. Rybakov, F. Theis, P. Angerer, F. A. Wolf (2024) anndata: Access and store annotated data matrices. (Zenodo).
26. J. D. Hunter, Matplotlib: A 2D graphics environment. *Computing in Science & Engineering* **9**, 90–95 (2007).
27. F. A. Wolf, P. Angerer, F. J. Theis, SCANPY: large-scale single-cell gene expression data analysis. *Genome Biol* **19**, 15 (2018).

28. M. Waskom, seaborn: statistical data visualization. *Journal of Open Source Software* **6** (2021).
29. H. Li *et al.*, The Sequence Alignment/Map format and SAMtools. *Bioinformatics* **25**, 2078–2079 (2009).
30. A. R. Quinlan, I. M. Hall, BEDTools: a flexible suite of utilities for comparing genomic features. *Bioinformatics* **26**, 841–842 (2010).
31. J. R. Conway, A. Lex, N. Gehlenborg, UpSetR: an R package for the visualization of intersecting sets and their properties. *Bioinformatics* **33**, 2938–2940 (2017).
32. M. Morgan, H. Pagès, V. Obenchain, N. Hayden (2025) Rsamtools: Binary alignment (BAM), FASTA, variant call (BCF), and tabix file import.
33. H. Wickham, J. Bryan (2025) readxl: Read Excel Files.
34. Team TBD (2021) BSgenome.Mmusculus.UCSC.mm10: Full genome sequences for Mus musculus (UCSC version mm10, based on GRCm38.p6).
35. H. Pagès (2025) BSgenome: Software infrastructure for efficient representation of full genomes and their SNPs.
36. M. Morgan, M. Lawrence, D. Van Twisk (2025) BiocIO: Standard Input and Output for Bioconductor Packages.
37. H. Pagès, P. Aboyoun, R. Gentleman, S. DebRoy (2025) Biostrings: Efficient manipulation of biological strings.
38. H. Pagès, P. Aboyoun (2025) XVector: Foundation of external vector representation and manipulation in Bioconductor.
39. J. Rainer (2017) EnsDb.Mmusculus.v79: Ensembl based annotation package.
40. J. Rainer, L. Gatto, C. X. Weichenberger, ensembldb: an R package to create and use Ensembl-based annotation resources. *Bioinformatics* **35**, 3151–3153 (2019).
41. M. Morgan, J. Rainer (2025) AnnotationFilter: Facilities for Filtering Bioconductor Annotation Resources.
42. M. Lawrence *et al.*, Software for computing and annotating genomic ranges. *PLoS Comput Biol* **9**, e1003118 (2013).
43. R. Koenker, P. Ng, SparseM: A Sparse Matrix Package for R *. *Journal of Statistical Software* **8**, 1–9 (2003).
44. H. Pagès, M. Carlson, S. Falcon, N. Li (2025) AnnotationDbi: Manipulation of SQLite-based annotations in Bioconductor.
45. R. Gentleman *et al.* (2025) graph: graph: A package to handle graph data structures.
46. M. E. Ritchie *et al.*, limma powers differential expression analyses for RNA-sequencing and microarray studies. *Nucleic Acids Res* **43**, e47 (2015).
47. H. Wickham (2025) stringr: Simple, Consistent Wrappers for Common String Operations.
48. H. Wickham, R. François, L. Henry, K. Müller, D. Vaughan (2026) dplyr: A Grammar of Data Manipulation.
49. H. Wickham, J. Hester, J. Bryan (2026) readr: Read Rectangular Text Data.
50. K. Müller, H. Wickham (2026) tibble: Simple Data Frames.
51. H. Wickham, D. Vaughan, M. Girlich, tidyr: Tidy Messy Data. (2026).
52. Y. Lin *et al.*, scMerge leverages factor analysis, stable expression, and pseudoreplication to merge multiple single-cell RNA-seq datasets. *Proc Natl Acad Sci U S A* **116**, 9775–9784 (2019).
53. A. T. Lun, D. J. McCarthy, J. C. Marioni, A step-by-step workflow for low-level analysis of single-cell RNA-seq data with Bioconductor. *F1000Res* **5**, 2122 (2016).
54. D. J. McCarthy, K. R. Campbell, A. T. Lun, Q. F. Wills, Scater: pre-processing, quality control, normalization and visualization of single-cell RNA-seq data in R. *Bioinformatics* **33**, 1179–1186 (2017).
55. G. Finak *et al.*, MAST: a flexible statistical framework for assessing transcriptional changes and characterizing heterogeneity in single-cell RNA sequencing data. *Genome Biol* **16**, 278 (2015).
56. R. A. Amezquita *et al.*, Orchestrating single-cell analysis with Bioconductor. *Nat Methods* **17**, 137–145 (2020).
57. M. Morgan, V. Obenchain, J. Hester, H. Pagès (2025) SummarizedExperiment: A container (S4 class) for matrix-like assays.
58. W. Huber *et al.*, Orchestrating high-throughput genomic analysis with Bioconductor. *Nat Methods* **12**, 115–121 (2015).
59. S. Arora, M. Morgan, M. Carlson, H. Pagès (2025) GenomeInfoDb: Utilities for manipulating chromosome names, including modifying them to follow a particular naming style.

60. H. Pagès, M. Lawrence, P. Aboyoun (2025) S4Vectors: Foundation of vector-like and list-like containers in Bioconductor.
61. C. Ahlmann-Eltze, P. Hickey, H. Pagès, MatrixGenerics: S4 Generic Summary Statistic Functions that Operate on Matrix-Like Objects. 10.18129/B9.bioc.MatrixGenerics (2025).
62. H. Bengtsson (2025) matrixStats: Functions that Apply to Rows and Columns of Matrices (and to Vectors).
63. T. Pedersen (2025) patchwork: The Composer of Plots.
64. K. Slowikowski, ggrepel: Automatically Position Non-Overlapping Text Labels with 'ggplot2'. (2025).
65. H. Wickham, *ggplot2: Elegant Graphics for Data Analysis* (Springer-Verlag, New York, 2016).
66. J. Baglama, L. Reichel, Augmented Implicitly Restarted Lanczos Bidiagonalization Methods. *SIAM Journal on Scientific Computing* **27**, 19–42 (2005).
67. R. Satija, A. Butler, P. Hoffman, T. Stuart, SeuratObject: Data Structures for Single Cell Data. (2023).
68. E. Pebesma, R. Bivand, Classes and methods for spatial data in R. *R News* **5**, 9–13 (2005).
69. R. S. Bivand, E. Pebesma, V. Gómez-Rubio, *Applied Spatial Data Analysis with R* (Springer, New York, NY, ed. Second, 2013), 10.1007/978-1-4614-7618-4.
70. S. Heinz *et al.*, Simple combinations of lineage-determining transcription factors prime cis-regulatory elements required for macrophage and B cell identities. *Mol Cell* **38**, 576–589 (2010).
71. J. T. Robinson *et al.*, Integrative genomics viewer. *Nat Biotechnol* **29**, 24–26 (2011).
72. W. N. Jefferson *et al.*, Widespread enhancer activation via ERalpha mediates estrogen response in vivo during uterine development. *Nucleic Acids Res* **46**, 5487–5503 (2018).
